# Supplementary material for: isoCirc catalogs full-length circular RNA isoforms in human transcriptomes
Source: Nat Commun. 2021 Jan 12;12:266. doi: 10.1038/s41467-020-20459-8 (PMC7803736; doi:10.1038/s41467-020-20459-8)
Supplement: Supplementary file 1 — Supplementary Information [file 41467_2020_20459_MOESM1_ESM.pdf]

Supplementary Fig. 1. Comparison of error rates for raw isoCirc reads and called consensus sequences across six HEK293 libraries.

Violin plots showing distributions of error rates for raw isoCirc reads and for consensus sequences called from 2, 3, 4, 5, 6 to 10, and more than 10 copies of a template circRNA sequence across six HEK293 libraries. For each boxplot, the middle horizontal line represents the median; the top and bottom horizontal lines of the rectangle represent the 3rd and 1st quartiles, respectively; the vertical line extends to the maximum and minimum values. Extreme outliers (error rates above 30%) were removed from plots. isoCirc reads that had consensus sequences called and were mappable to the human genome are represented in the plots. Plots show analyses of n = 2 biological replicates (each with n = 3 technical replicates).

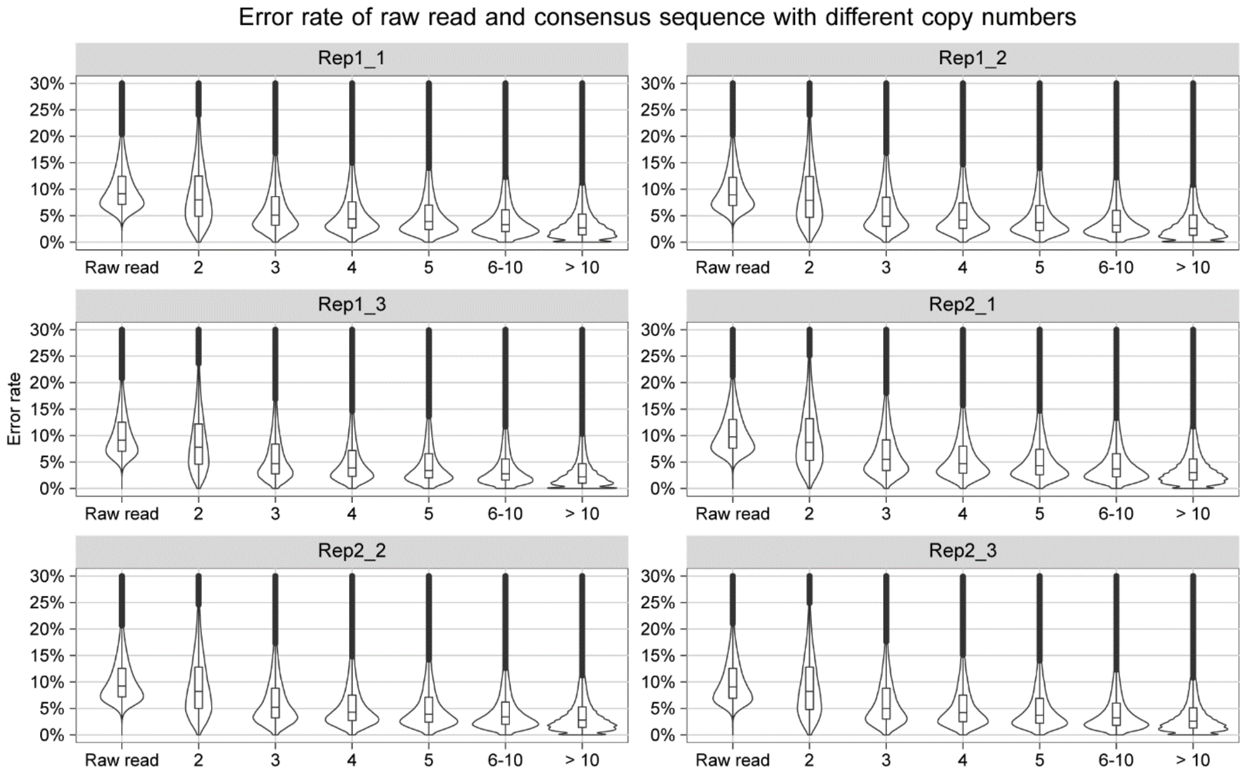

Supplementary Fig. 2. Comparison of raw isoCirc read lengths and consensus sequence lengths across six HEK293 libraries and 12 human tissues.

a Violin plots showing distributions of lengths (in bp) for raw isoCirc reads across six HEK293 libraries and 12 human tissues. isoCirc raw reads for which consensus sequences were called and high-confidence BSJs were detected are represented in the plots.

b Violin plots showing distributions of lengths (in bp) for called consensus sequences across six HEK293 libraries and 12 human tissues. Consensus sequences for which high-confidence BSJs were detected are represented in the plots.

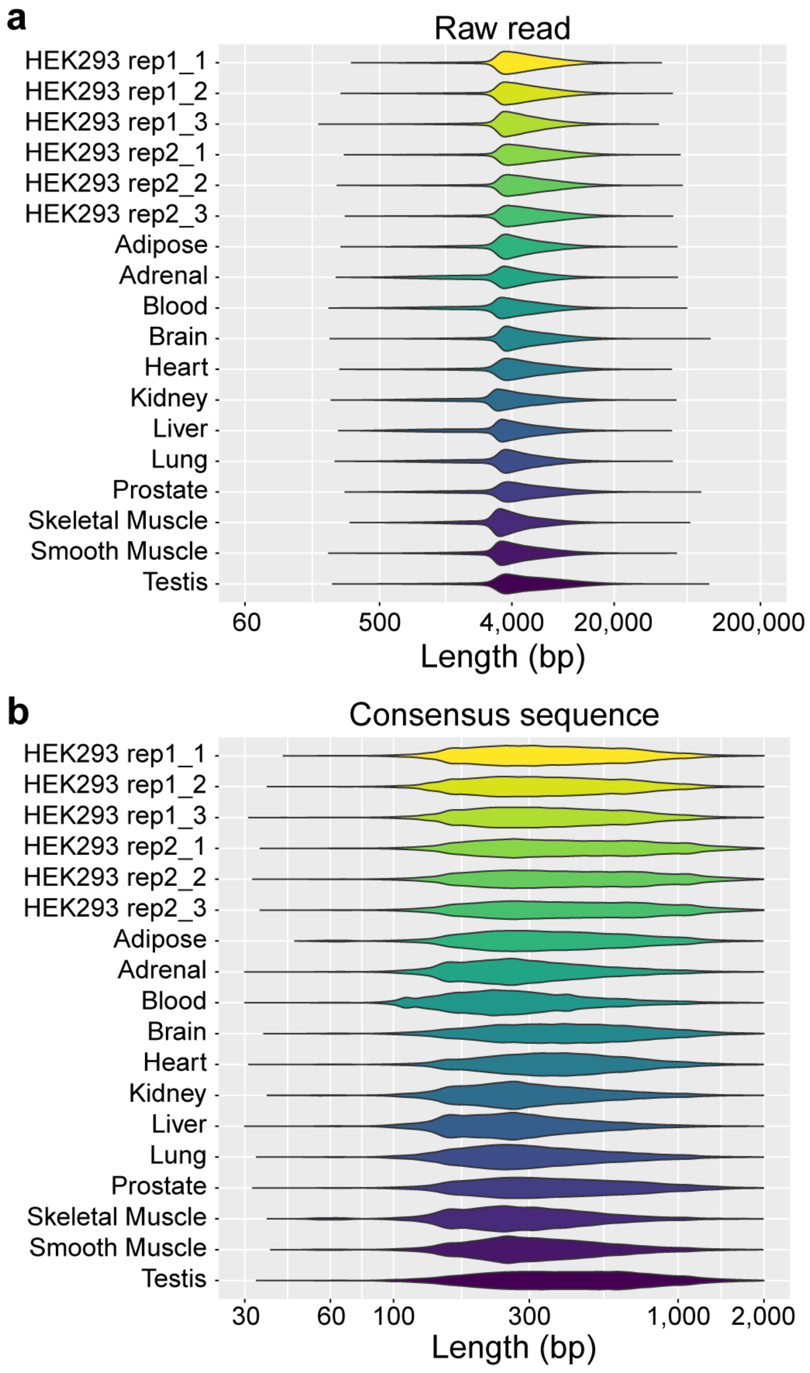

Supplementary Fig. 3. Pairwise comparisons of similarity between high-confidence BSJs identified from six HEK293 libraries, at various read-count thresholds.

Heatmaps showing pairwise comparisons of similarity between high-confidence BSJs identified from six HEK293 libraries. For each library pair, the degree of similarity was calculated as the number of shared high-confidence BSJs found in both libraries, divided by the total number of high-confidence BSJs in either library. Color reflects the degree of similarity between two libraries, as indicated by the legend. BSJs with read count ≥ 1 (top), read count ≥ 2 (middle), or read count ≥ 3 (bottom) were included in separate plots.

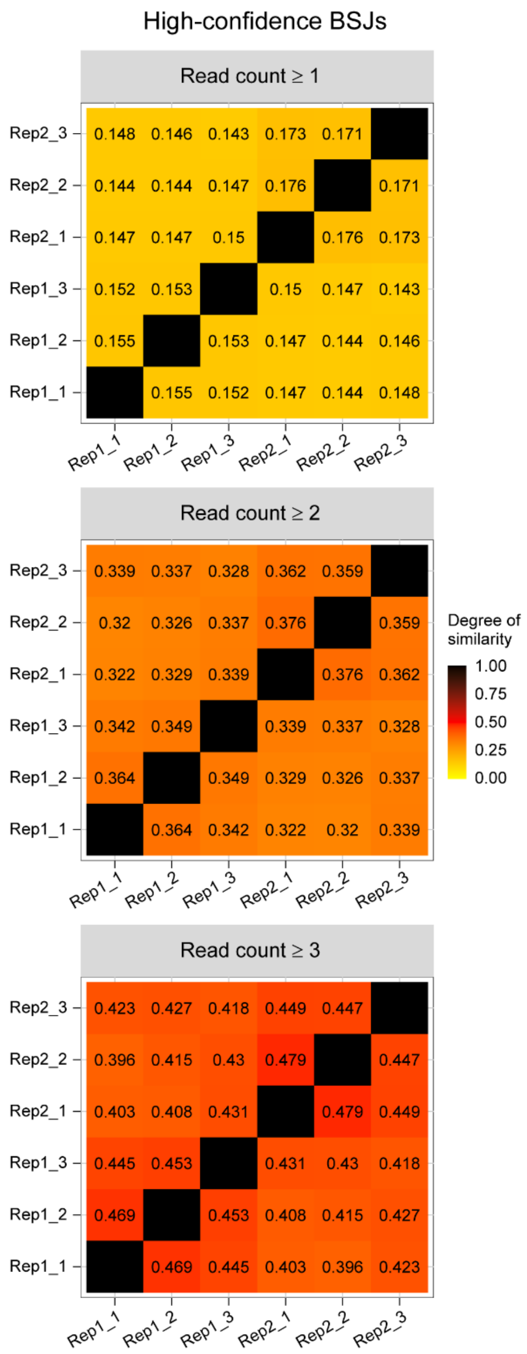

Supplementary Fig. 4. Pairwise comparisons of similarity between full-length circRNA isoforms identified from six HEK293 libraries, at various read-count thresholds.
Heatmaps showing pairwise comparisons of similarity between full-length circRNA isoforms identified from six HEK293 libraries. For each library pair, the degree of similarity was calculated as the number of shared full-length circRNA isoforms found in both libraries, divided by the total number of full-length circRNA isoforms in either library. Color reflects the degree of similarity between two libraries, as indicated by the legend. Full-length circRNA isoforms with read count ≥ 1 (top), read count ≥ 2 (middle), or read count ≥ 3 (bottom) were included in separate plots.

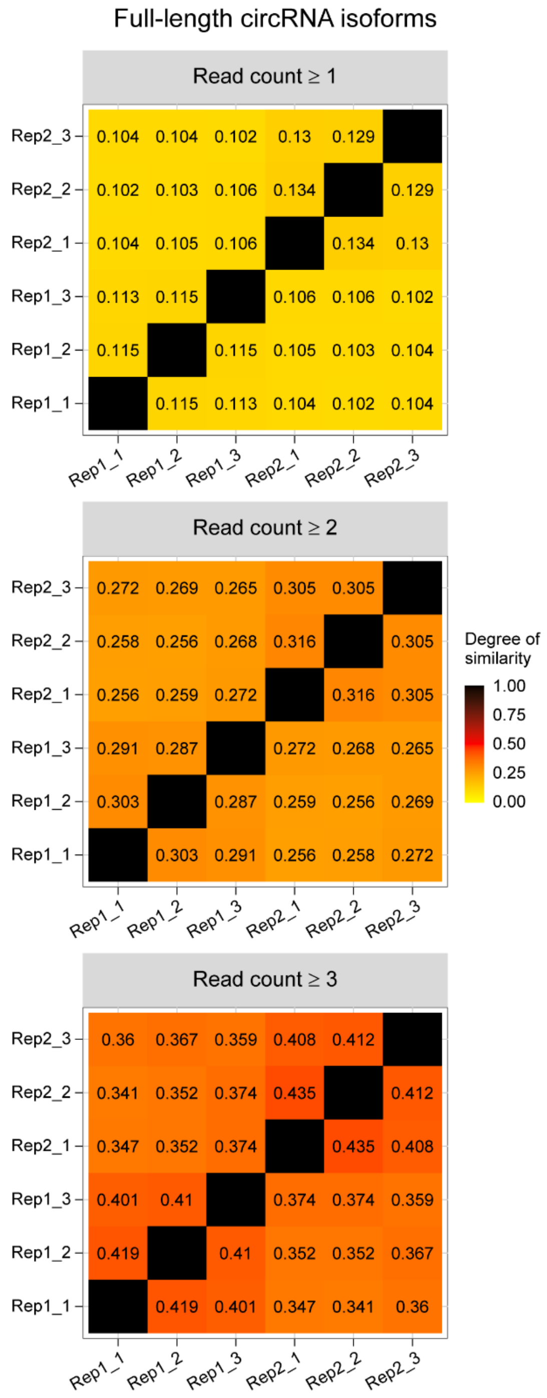

Supplementary Fig. 5. Reproducibility of isoCirc data based on high-confidence BSJ read count across six HEK293 libraries.

Scatter plots showing pairwise comparisons of isoCirc read counts for high-confidence BSJs identified from six HEK293 libraries. Plots show analyses of n = 2 biological replicates (each with n = 3 technical replicates). For each scatter plot, the *x*-axis shows the isoCirc read counts of high-confidence BSJs from the library labeled on the top. The *y*-axis shows the isoCirc read counts of high-confidence BSJs from the library labeled on the right. In each pairwise comparison, BSJs with read count ≥ 1 in at least one library were included in the plot.

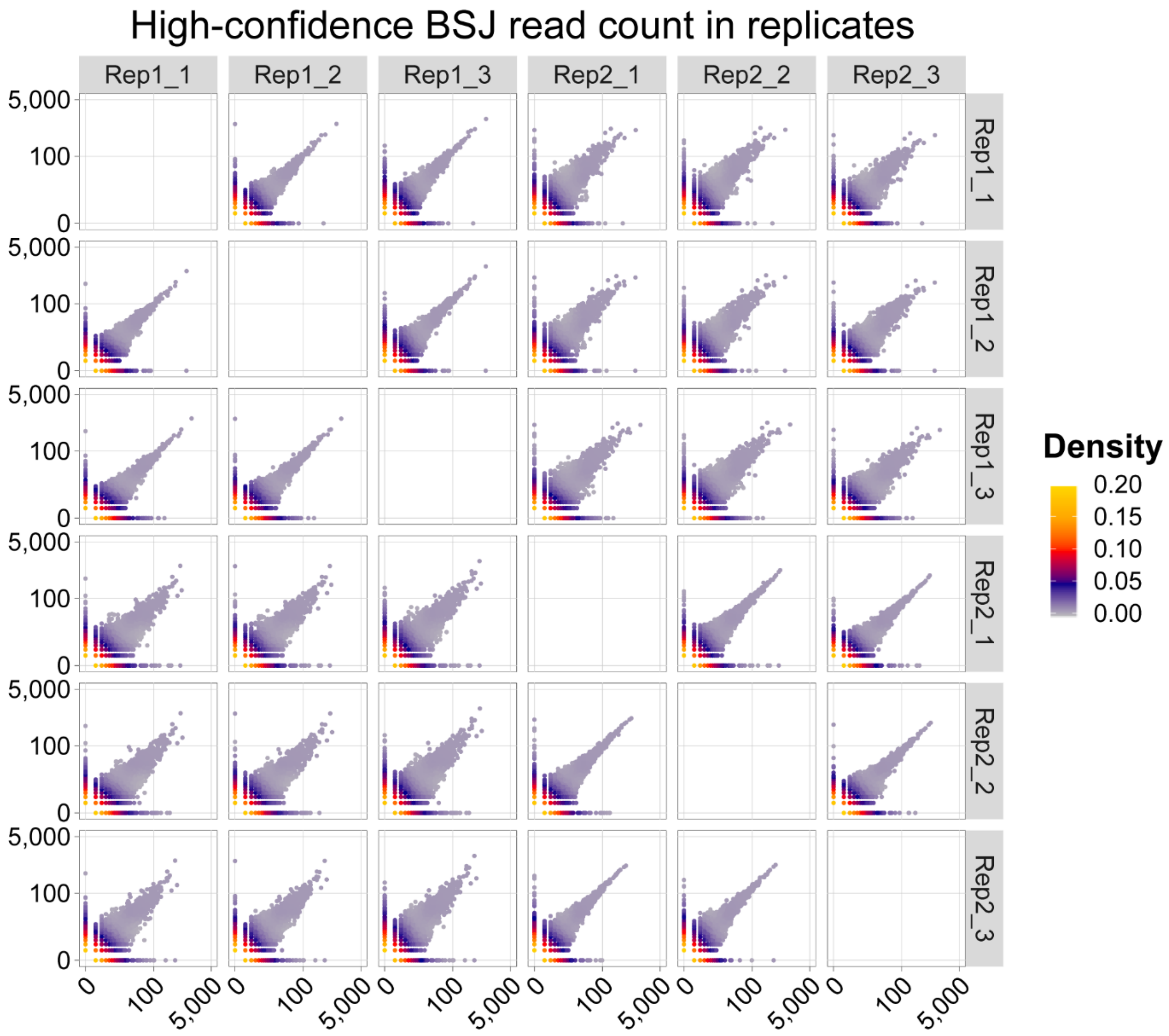

Supplementary Fig. 6. Reproducibility of isoCirc data based on full-length circRNA isoform read count across six HEK293 libraries.

Scatter plots showing pairwise comparisons of isoCirc read counts for full-length circRNA isoforms identified from six HEK293 libraries. Plots show analyses of n = 2 biological replicates (each with n = 3 technical replicates). For each scatter plot, the *x*-axis shows the isoCirc read counts of full-length circRNA isoforms from the library labeled on the top. The *y*-axis shows the isoCirc read counts of full-length circRNA isoforms from the library labeled on the right. In each pairwise comparison, full-length circRNA isoforms with read count ≥ 1 in at least one library were included in the plot.

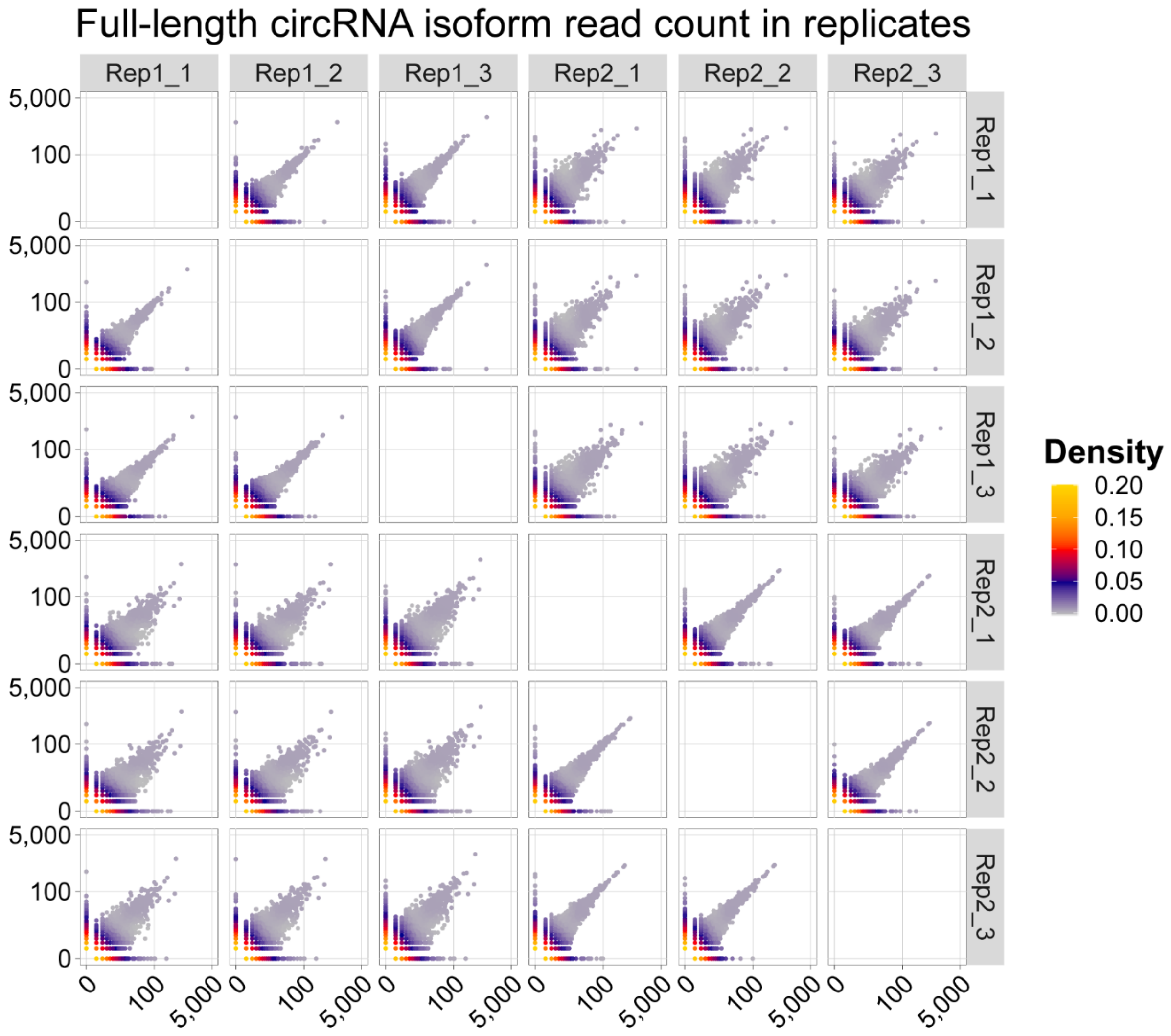

Supplementary Fig. 7. Proportion of circRNA reads across different types of short-read and isoCirc long-read libraries of HEK293 cells.

Barplots showing proportions of reads with circRNA BSJs across different types of short-read and isoCirc long-read libraries. Data are shown for three poly(A)-selected Illumina short-read libraries and three RNase R-treated Illumina short-read libraries, as well as six isoCirc long-read libraries (two biological replicates: isoCirc biorep1/2, each with three technical replicates). For each library, the number of reads with identified circRNA BSJs was divided by the total number of reads in that library, to represent the percentage of circRNA reads in that library.

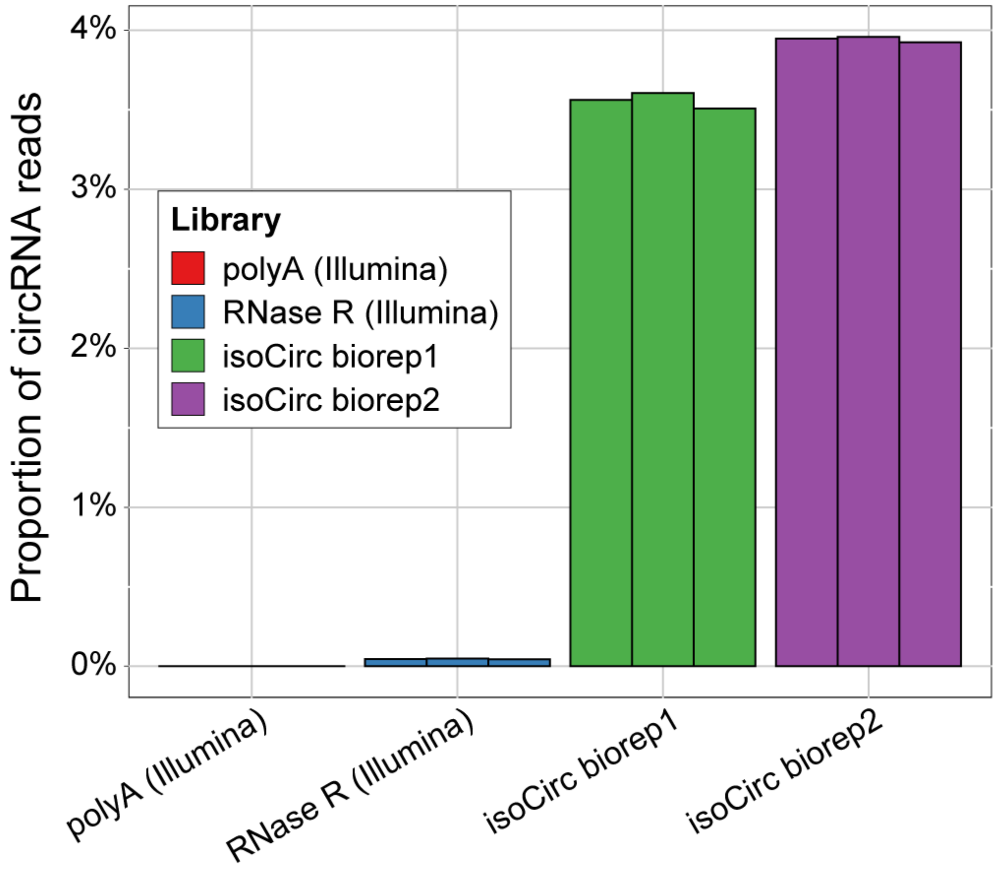

Supplementary Fig. 8. Pairwise comparisons of similarity between BSJs identified from three RNase R-treated Illumina RNA-seq libraries, at various read-count thresholds. Heatmap showing pairwise comparisons of similarity between BSJs identified from three RNase R-treated Illumina RNA-seq libraries. For each library pair, the degree of similarity was calculated as the number of shared BSJs found in both libraries, divided by the total number of BSJs in either library. Color reflects the degree of similarity between two libraries, as indicated by the legend. BSJs with read count ≥ 2 (top) or read count ≥ 3 (bottom) were included in separate plots.

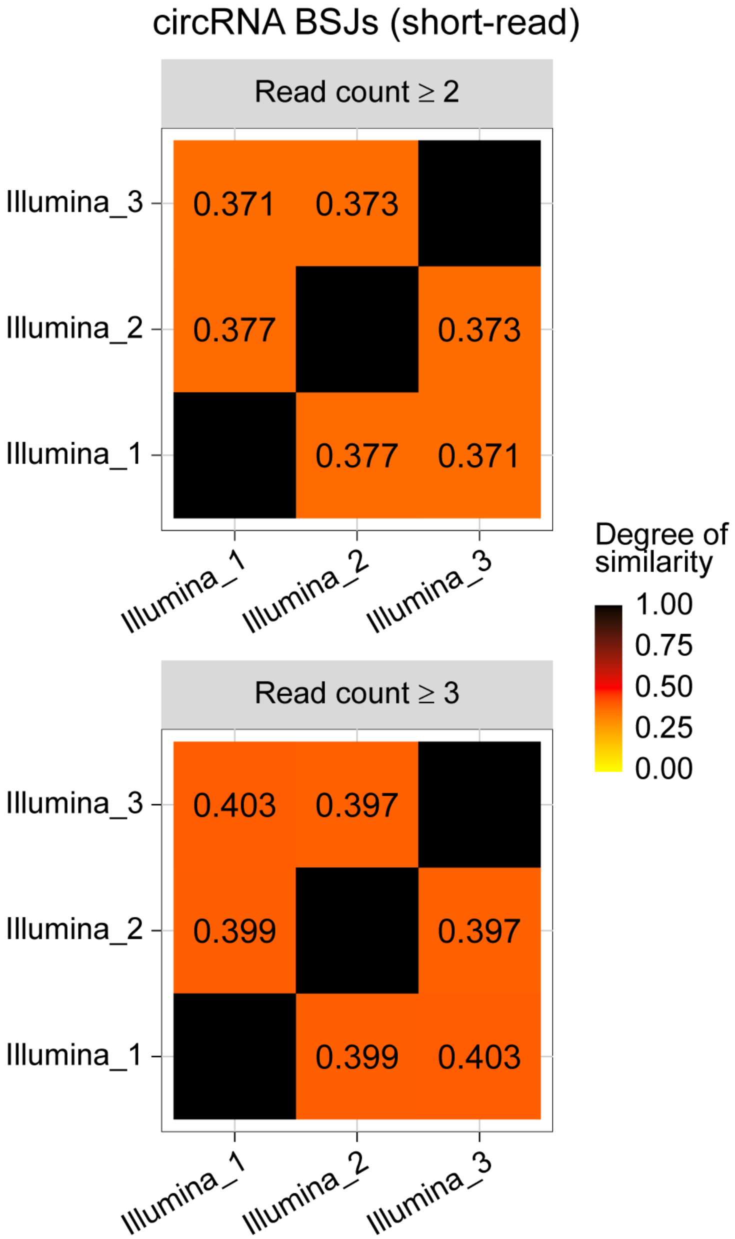

Supplementary Fig. 9. Comparison of high-confidence BSJ read counts between short-read and isoCirc long-read datasets of HEK293 cells.

Scatter plot showing the correlation of circRNA read counts between RNase R-treated short-read libraries (n = 3 biological replicates) and isoCirc long-read libraries of HEK293 cells (n = 2 biological replicates, each with n = 3 technical replicates), for high-confidence BSJs identified from both short-read and isoCirc long-read data. For a given BSJ, its read counts were summed over individual replicates of short-read or isoCirc long-read libraries, respectively. BSJs with ≥ 2 reads in both short-read and isoCirc long-read data were included in the plot.

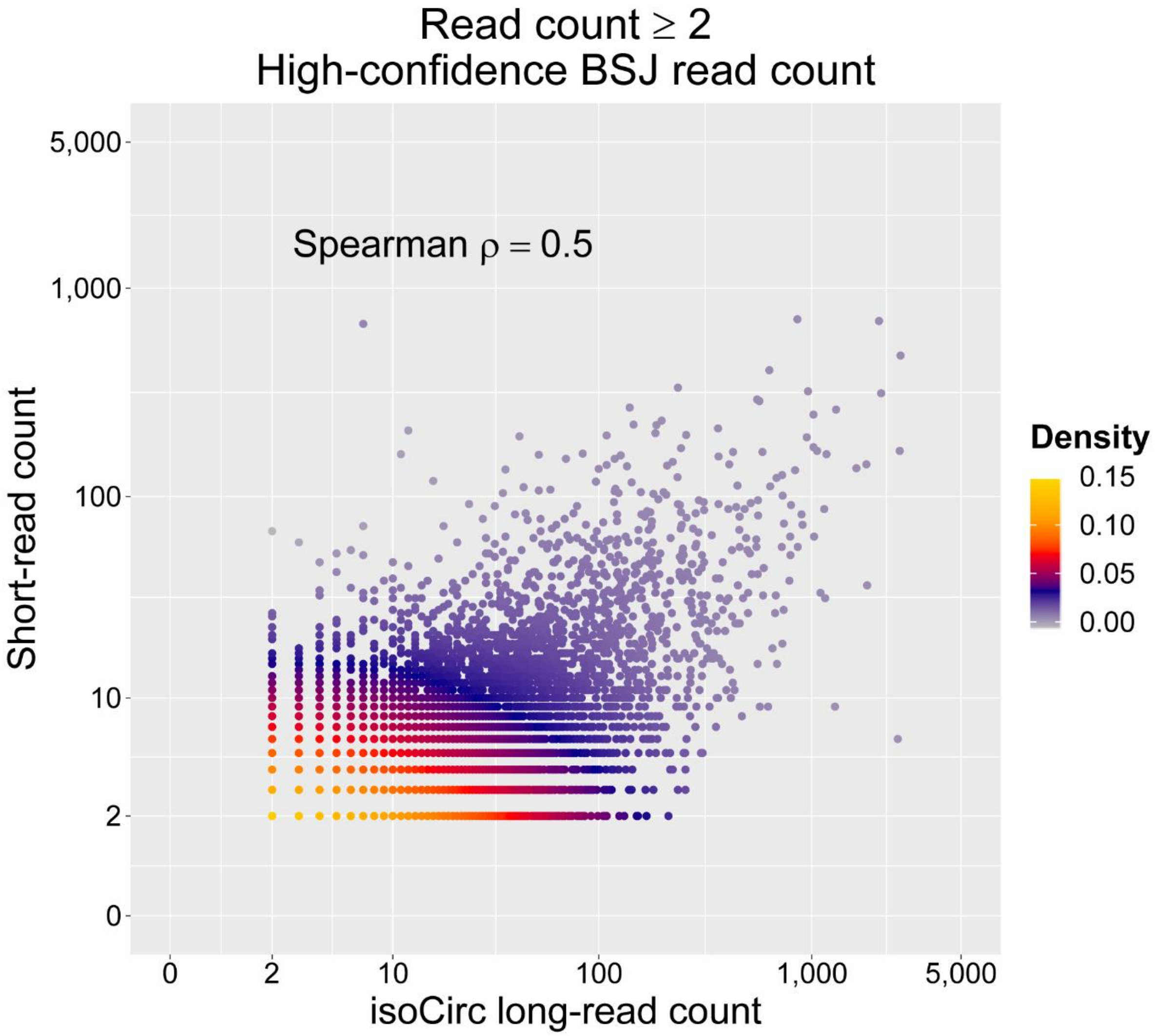

Supplementary Fig. 10. Fraction of known or novel high-confidence BSJs with read count ≥ 3 identified in two biological replicates of HEK293 cells.
Stacked barplot showing fraction of known or novel high-confidence BSJs identified in only one ('1') or both ('2') biological replicates of HEK293 cells, based on BSJ annotations in circBase (http://www.circbase.org) and MiOncoCirc (https://mioncocirc.github.io) databases. Only BSJs with read count ≥ 3 in each biological replicate (summing over its 3 technical replicates) were included. Bars show known BSJs annotated in circBase only (red), MiOncoCirc only (blue), or both databases ('Both', green), and novel BSJs not annotated in either database ('Novel', purple).

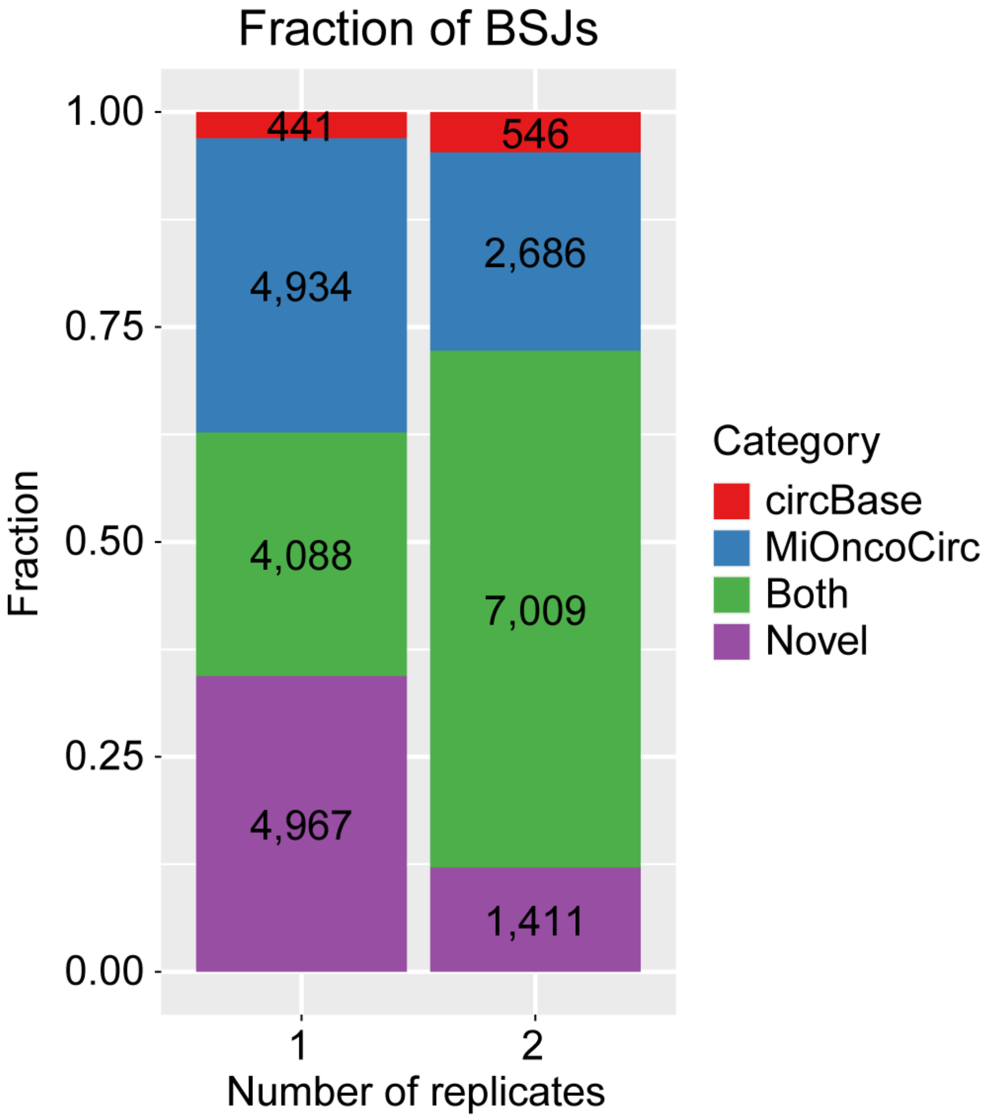

Supplementary Fig. 11. Cumulative distribution plots of read counts in 2 replicates for high-confidence BSJs identified in HEK293 cells.

A Cumulative distribution plots of read counts in 2 replicates for BSJs at read count ≥ 2. BSJs were classified based on their annotation status and level of detection across biological replicates of HEK293 cells, as follows: BSJ is known and detected in both replicates (red); BSJ is novel and detected in both replicates (blue); BSJ is known and detected in only one replicate (green); and BSJ is novel and detected in only one replicate (purple).

B Cumulative distribution plots of read counts in 2 replicates for BSJs at read count ≥ 3. Details are as in (**a**).

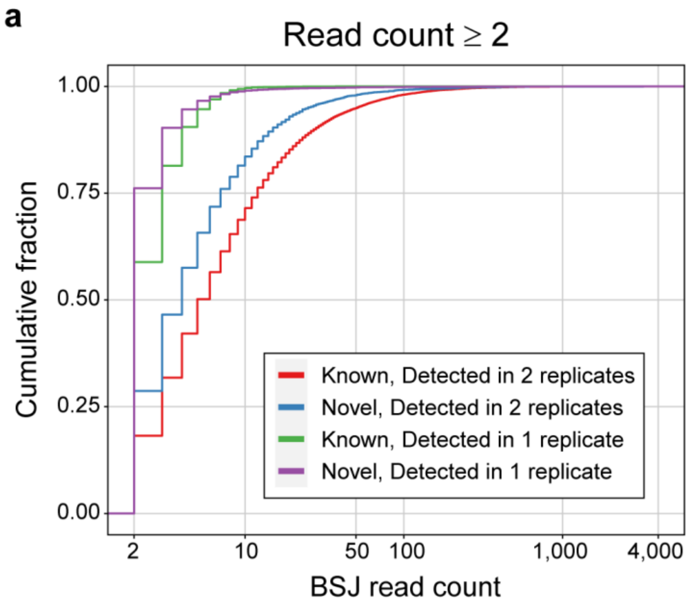

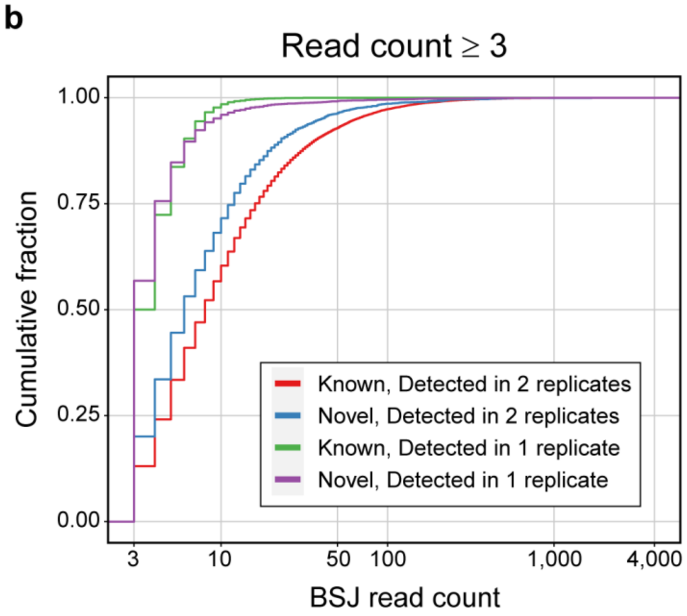

Supplementary Fig. 12. Fractions of known or novel BSJs identified by isoCirc with inverted Alu repeats in flanking introns.

Stacked barplots showing fractions of known or novel BSJs identified by isoCirc with inverted Alu repeats in flanking introns with window sizes 1,000 bp (top) and 2,000 bp (bottom). Inverted Alu repeats were classified based on their orientation as convergent (red), divergent (blue), both (green), or no inverted Alu repeats detected (purple). BSJs with read count ≥ 2 (left column) and read count ≥ 3 (right column) were classified based on their annotation status and level of detection across biological replicates of HEK293 cells, as follows: BSJ is novel and detected in only one replicate (1Novel); BSJ is novel and detected in both replicates (2Novel); BSJ is known and detected in only one replicate (1Known); and BSJ is known and detected in both replicates (2Known). A fifth set of negative-control BSJs (Negative) was constructed by making 10,000 random pairs of a downstream 5' splice site and an upstream 3' splice site, using non-BSJ splice sites based on isoCirc results and circRNA databases (circBase and MiOncoCirc).

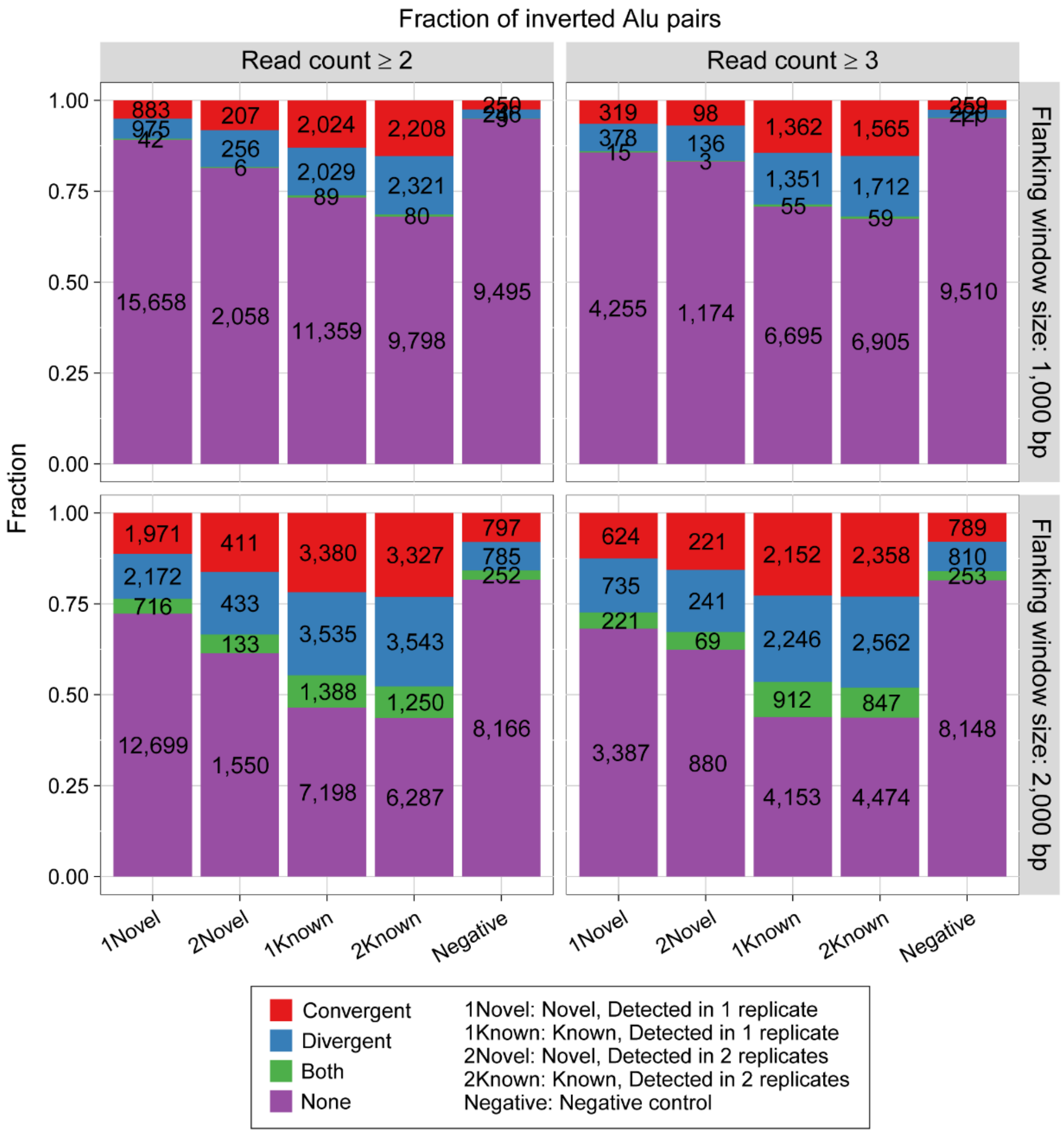

Supplementary Fig. 13. Numbers of full-length circRNA isoforms with read count ≥ 3 in six HEK293 libraries combined, based on BSJ and FSJ categories.

Heatmap showing numbers of full-length circRNA isoforms identified in HEK293 cells, based on their BSJ (*x*-axis) and FSJ (*y*-axis) categories, as classified relative to existing annotations of circRNA or linear RNA transcripts. Only full-length circRNA isoforms with read count ≥ 3 (summing over all six libraries) were included in the analysis. Three categories were used to classify all identified circRNA BSJs and FSJs: Full Splice Match (FSM), Novel In Catalog (NIC), and Novel Not in Catalog (NNC).

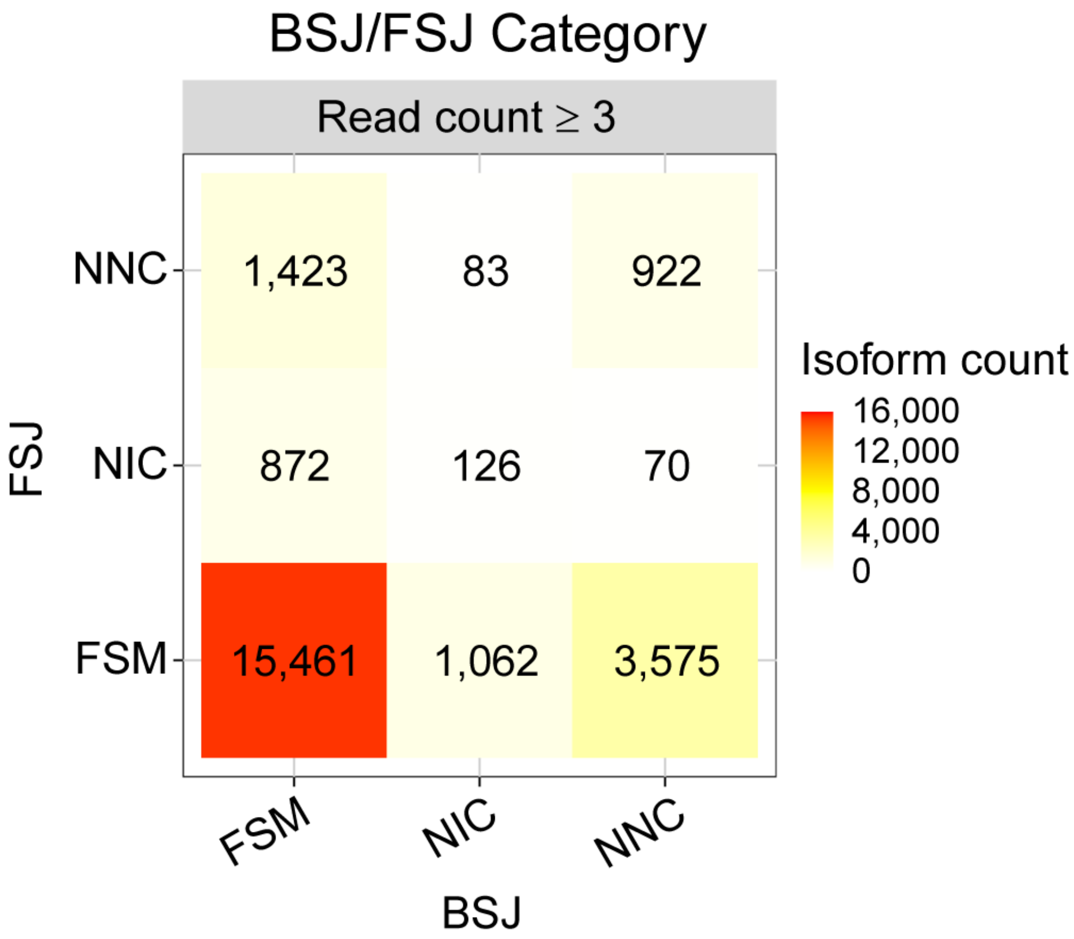

Supplementary Fig. 14. Transcript structures and supporting isoCirc consensus sequences for the four most abundant *KDM1A* circRNA isoforms in HEK293 cells.
isoCirc read coverage track for aggregated HEK293 replicates displaying the four most abundant *KDM1A* circRNA isoforms (KDM1A.circRNA.1, KDM1A.circRNA.5, KDM1A.circRNA.4, KDM1A.circRNA.7). A separate track displaying base-level conservation scores across vertebrates (phyloP 46-way) is also supplied. Transcript structures and BSJs of isoforms are shown using red boxes and black arrows. Total number of reads across HEK293 replicates for each isoform is indicated next to the isoform identifier. Supporting isoCirc consensus sequences for the four isoforms are also displayed, with grey, purple, and blue segments representing aligned regions, insertions, and gaps, respectively, relative to the reference genome.

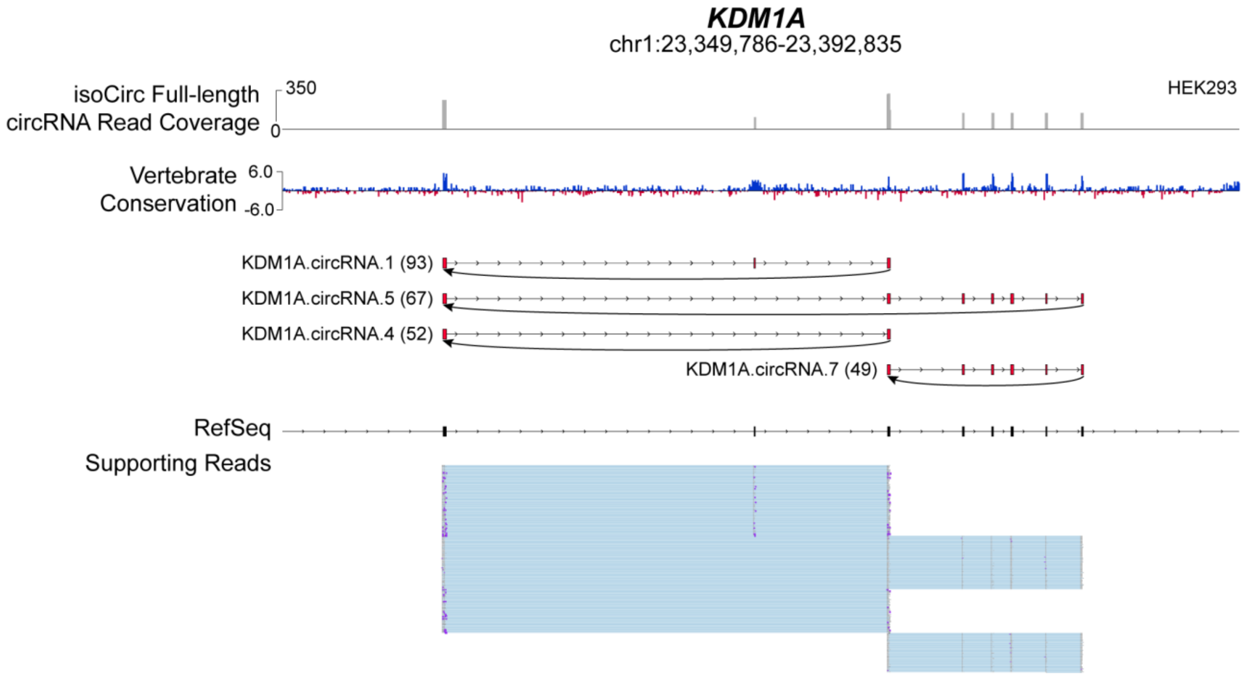

Supplementary Fig. 15. Cumulative distribution plots of isoCirc read count of full-length circRNA isoforms identified from isoCirc data of 12 human tissues.

Cumulative distribution plots of isoCirc read count of full-length circRNA isoforms with read count ≥ 2 in each of 12 human tissues. Isoforms were classified by their BSJ-FSJ categories, as follows: both BSJ and FSJ were FSM or NIC (red: FSM/NIC-FSM/NIC); BSJ was FSM or NIC, FSJ was NNC (blue: FSM/NIC-NNC); BSJ was NNC, FSJ was FSM or NIC (green: NNC-FSM/NIC); both BSJ and FSJ were NNC (purple: NNC-NNC); and all isoforms were combined (black: All-All).

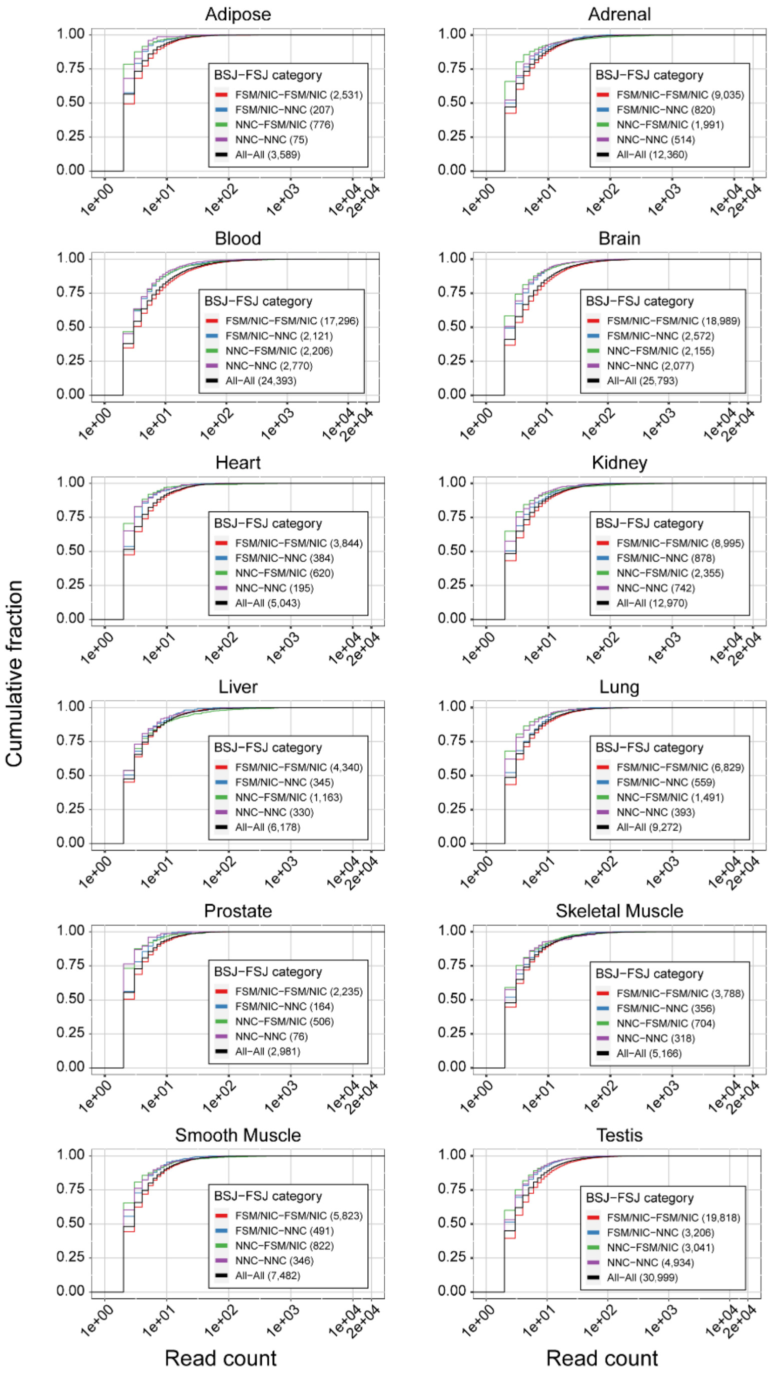

Supplementary Fig. 16. Numbers of full-length circRNA isoforms with read count ≥ 2 in at least one of 12 human tissues, based on BSJ and FSJ categories.

Heatmap showing numbers of full-length circRNA isoforms identified in 12 human tissues, based on their BSJ (*x*-axis) and FSJ (*y*-axis) categories, as classified relative to existing annotations of circRNA or linear RNA transcripts. Only full-length circRNA isoforms with read count ≥ 2 in at least one tissue were included in the analysis. Three categories were used to classify all identified circRNA BSJs and FSJs: Full Splice Match (FSM), Novel In Catalog (NIC), and Novel Not in Catalog (NNC).

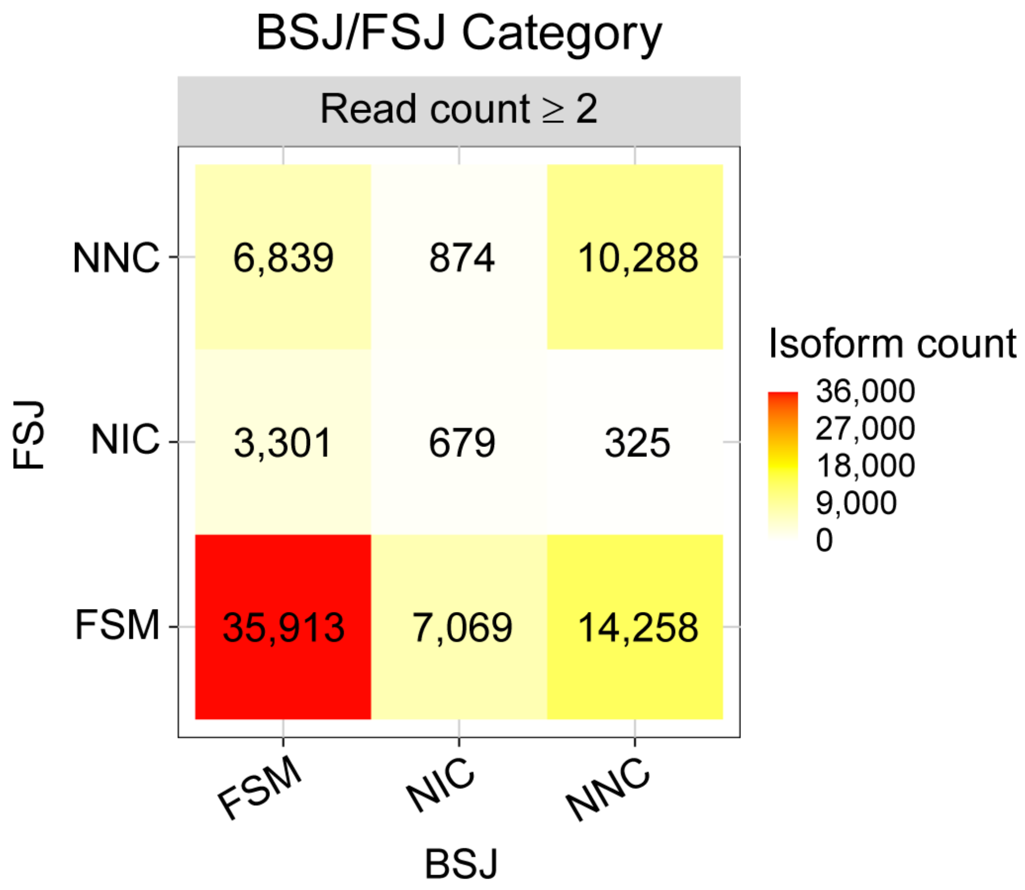

Supplementary Fig. 17. Cumulative distribution plots of full-length circRNA isoform number per gene or per BSJ in human tissues, for circRNA isoforms with read count ≥ 2 in at least one of 12 human tissues.
a Cumulative distribution plot of full-length circRNA isoform number per gene.
b Cumulative distribution plot of full-length circRNA isoform number per BSJ.

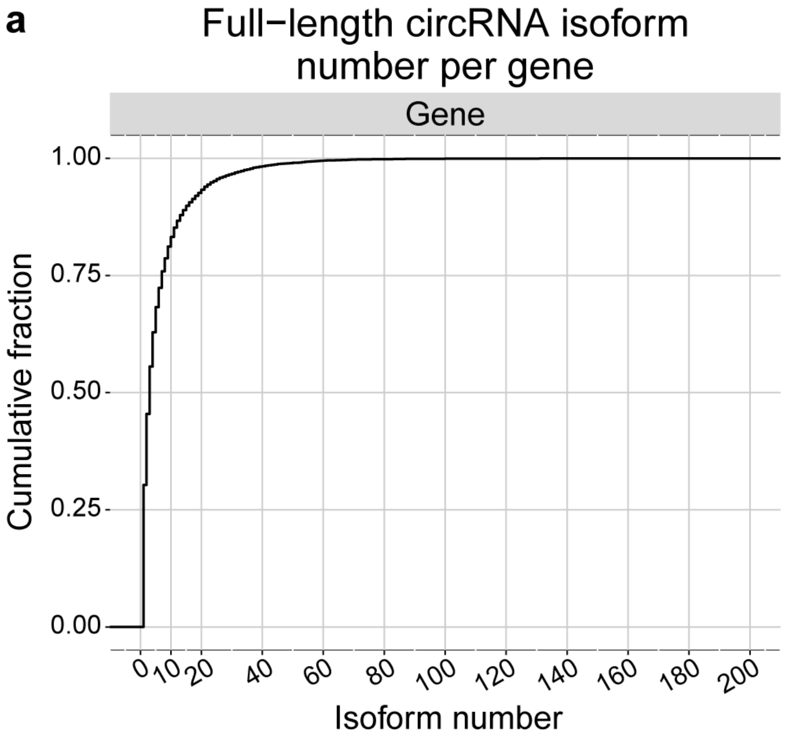

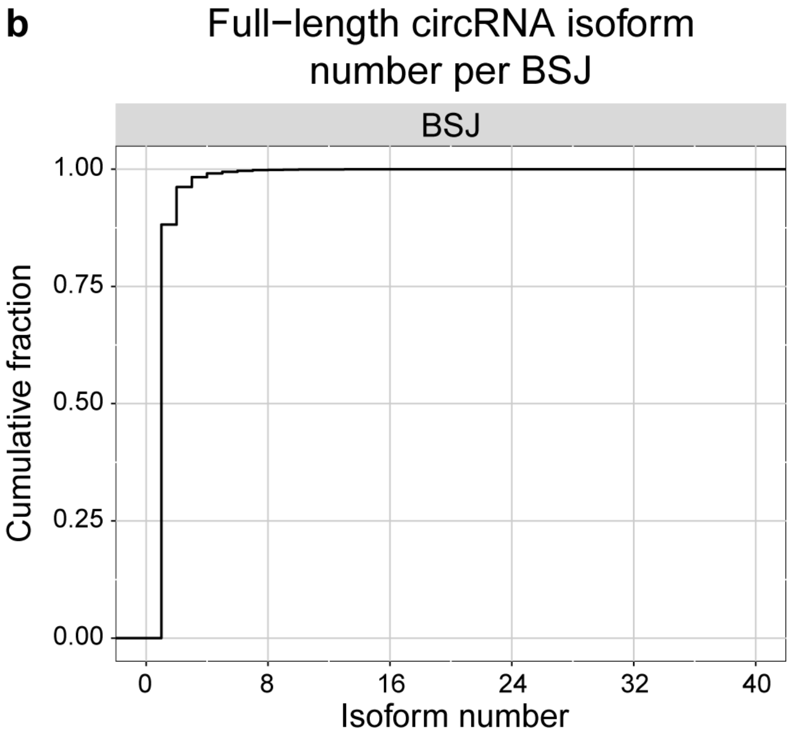

Supplementary Fig. 18. Pairwise comparison of circRNA isoform proportions across 12 human tissues.

Scatter plots showing pairwise comparisons of circRNA isoform proportions between each pair of 12 human tissues (n = 1 biological replicate; the total RNA sample of each human tissue was a pooled sample extracted from tissues of multiple donors, as described in Supplementary Table 4). Only circRNA isoforms with differential isoform proportions in a pairwise tissue comparison were included in the plot for that tissue pair. For each scatter plot, the *x*-axis shows the proportions of circRNA isoforms from the tissue labeled on the top. The *y*-axis shows the proportions of circRNA isoforms from the tissue labeled on the right.

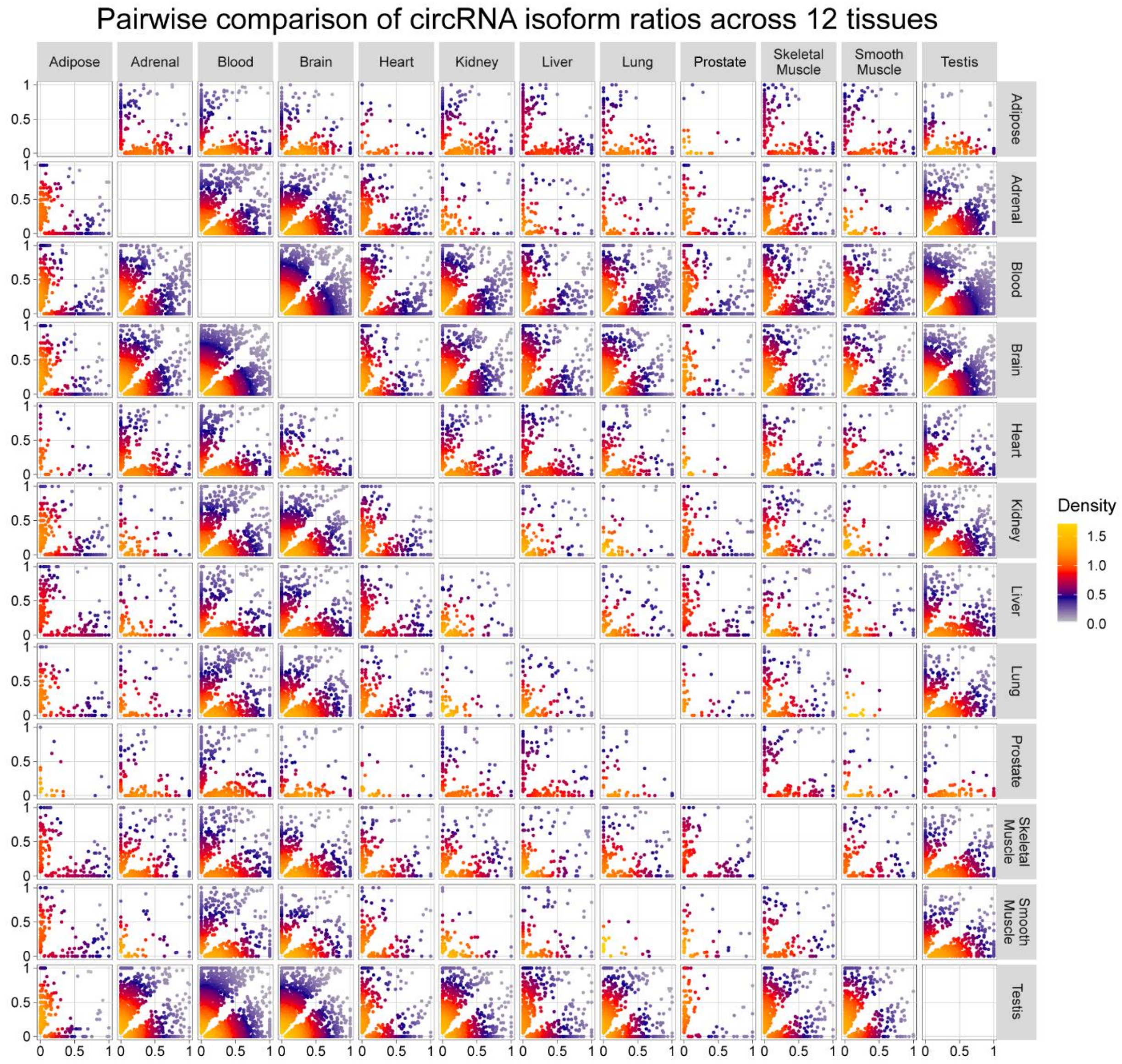

Supplementary Fig. 19. Stacked barplots showing isoform proportions of *UBE2G1* and *PDE8A* circRNA isoforms across 12 human tissues.

a Stacked barplot of isoform proportions of *UBE2G1* circRNA isoforms across 12 human tissues. CircRNA isoforms were included in the plot if read count was ≥2 in at least one tissue, and both BSJ and FSJs were FSM or NIC (FSM/NIC-FSM/NIC). Total read count for all circRNA isoforms in a tissue is given in parentheses on the *x*-axis.

b Stacked barplot of isoform proportions of *PDE8A* circRNA isoforms across 12 human tissues. Details for the plot are the same as in (**a**).

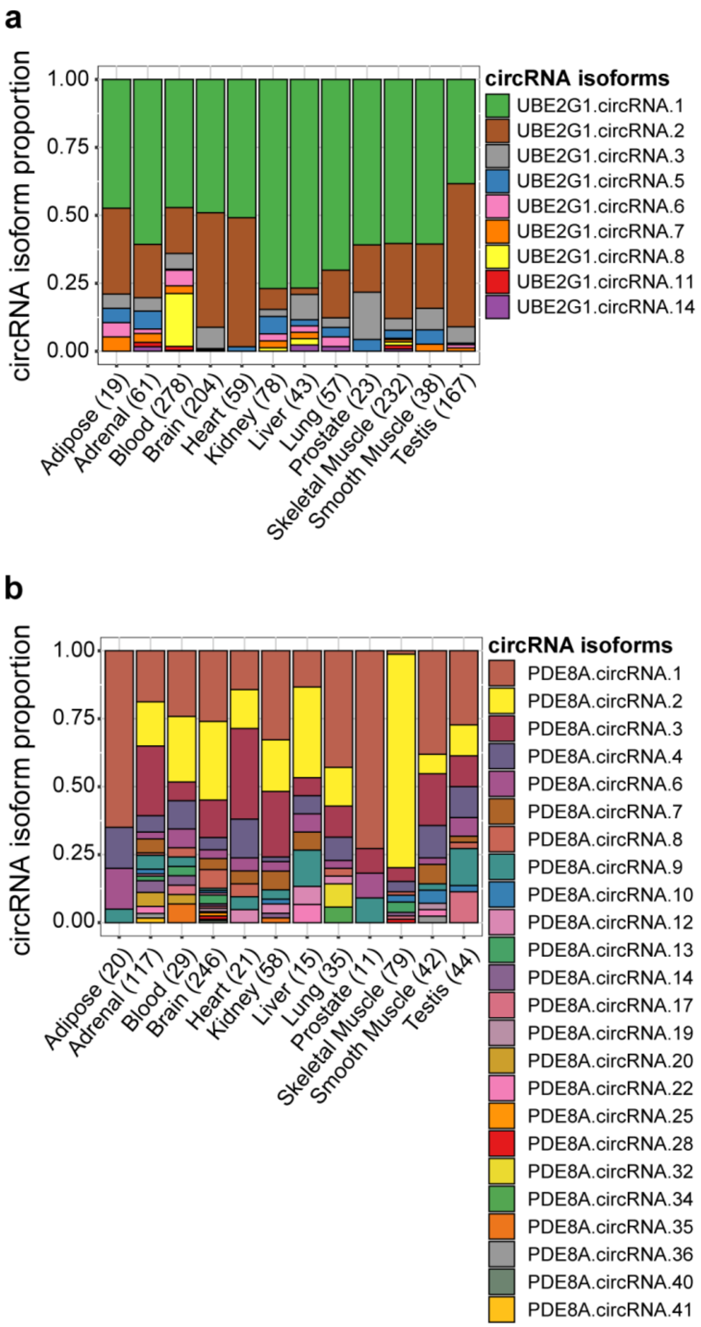

Supplementary Fig. 20. Stacked barplots showing isoform proportions of *HIPK3* and *UBXN7* circRNA isoforms across 12 human tissues.

a Stacked barplot of isoform proportions of *HIPK3* circRNA isoforms across 12 human tissues. CircRNA isoforms were included in the plot if read count was ≥2 in at least one tissue, and both BSJ and FSJs were FSM or NIC (FSM/NIC-FSM/NIC). Total read count for all circRNA isoforms in a tissue is given in parentheses on the *x*-axis.

b Stacked barplot of isoform proportions of *UBXN7* circRNA isoforms across 12 human tissues. Details for the plot are the same as in (**a**).

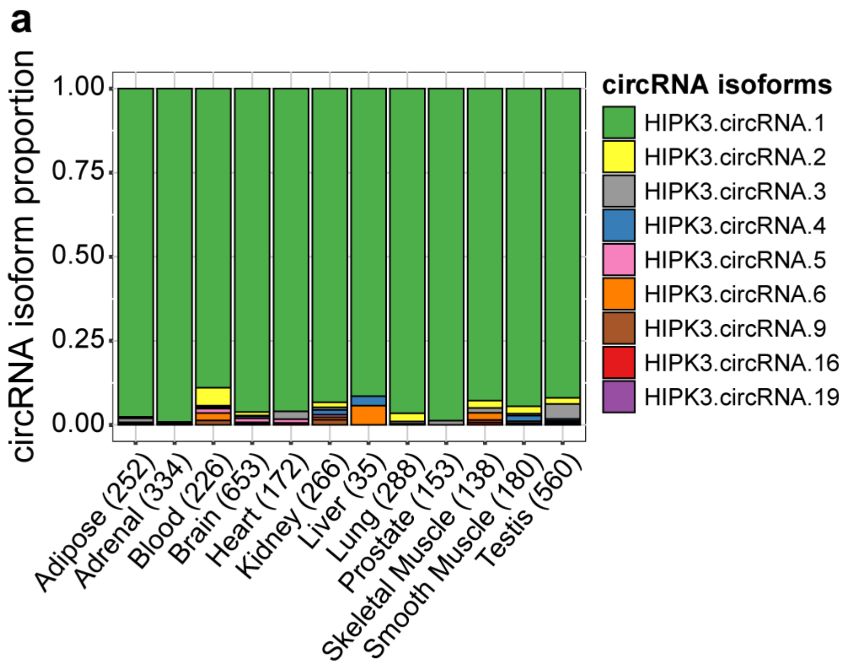

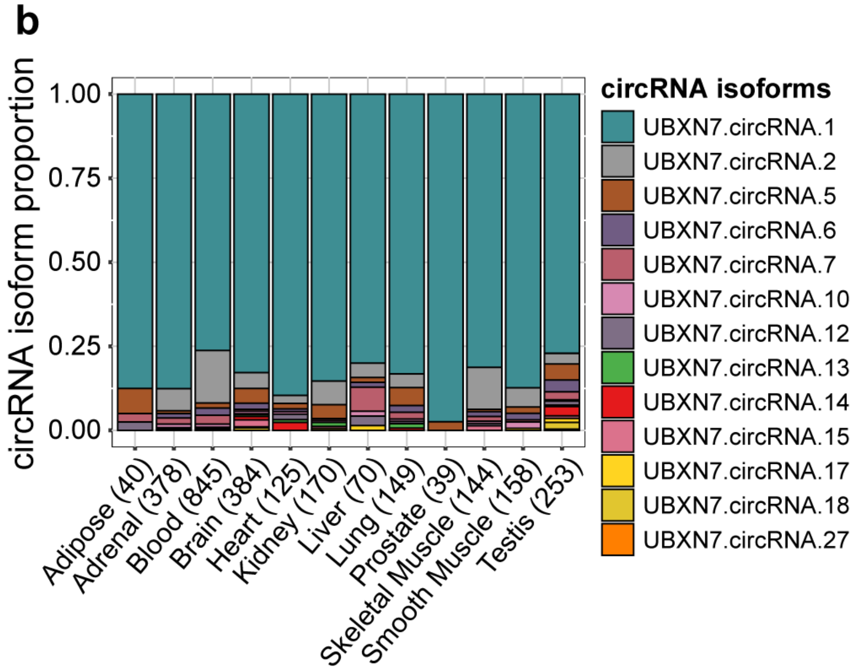

Supplementary Fig. 21. isoCirc reveals an internal alternative splicing event in circRNA isoforms of *RAB3IP* corresponding to a retained intron event.

isoCirc read coverage tracks for 12 human tissues and aggregated HEK293 replicates displaying two circRNA isoforms of *RAB3IP*, RAB3IP.circRNA.1 and RAB3IP.circRNA.4, which had a retained intron event between the two isoforms. A separate track displaying base-level conservation scores across vertebrates (phyloP 46-way) is also supplied. Transcript structures and BSJs of RAB3IP.circRNA.1 and RAB3IP.circRNA.4 are displayed using red boxes and black arrows. Total number of reads across all 12 human tissues and HEK293 replicates for each isoform is indicated next to the isoform identifier.

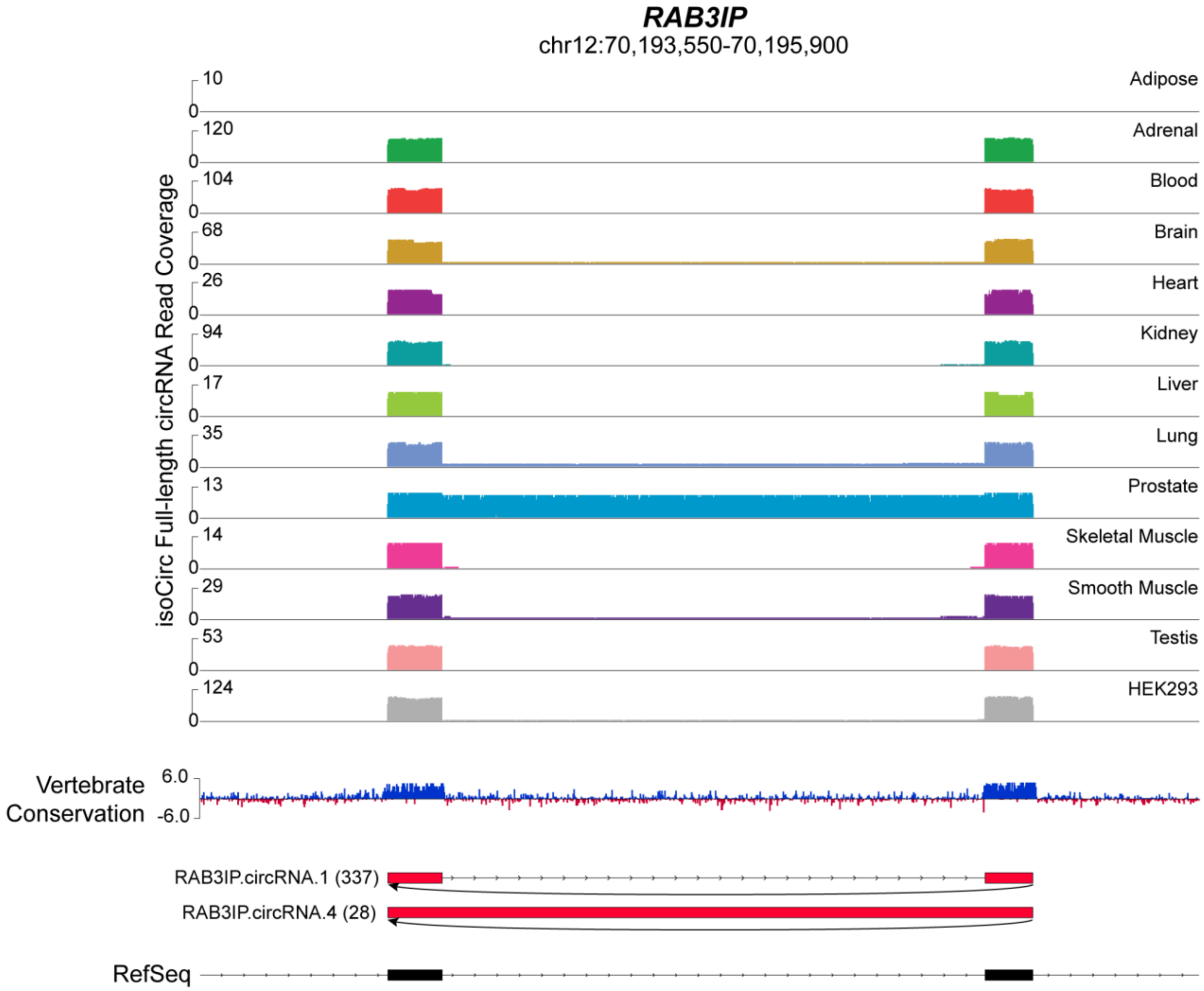

Supplementary Fig. 22. isoCirc reveals internal alternative splicing events in circRNA isoforms of *C2CD5* and *QKI* corresponding to skipped exon events.

a isoCirc read coverage tracks for 12 human tissues and aggregated HEK293 replicates displaying two circRNA isoforms of *C2CD5*, C2CD5.circRNA.1 and C2CD5.circRNA.4, which had a skipped exon event between the two isoforms. A separate track displaying base-level conservation scores across vertebrates (phyloP 46-way) is also supplied. Transcript structures and BSJs of C2CD5.circRNA.1 and C2CD5.circRNA.4 are displayed using red boxes and black arrows. Total number of reads across all 12 human tissues and HEK293 replicates for each isoform is indicated next to the isoform identifier.

b isoCirc read coverage tracks for 12 human tissues and aggregated HEK293 replicates displaying two circRNA isoforms of *QKI*, QKI.circRNA.1 and QKI.circRNA.2, which had a skipped exon event between the two isoforms. Details for the plot are the same as in (**a**).

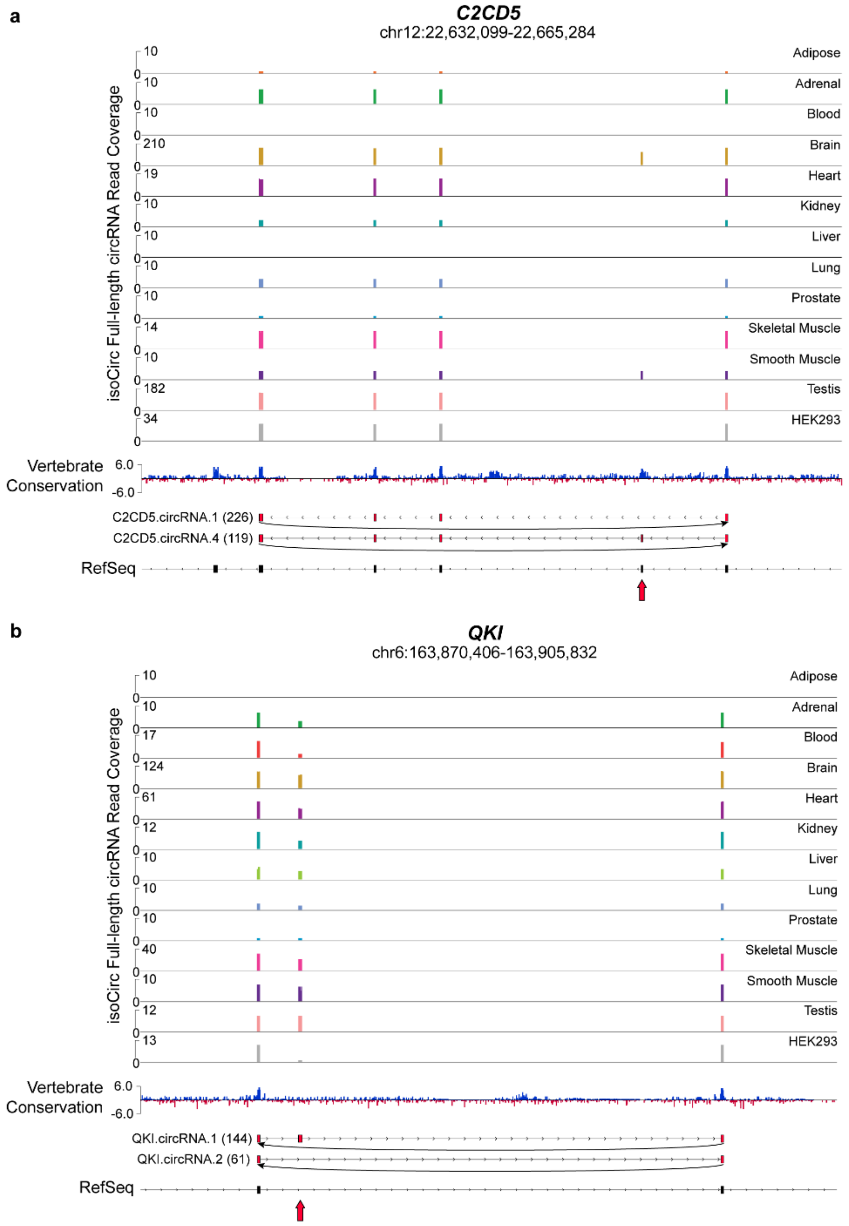

Supplementary Fig. 23. Schematic diagrams for identifying canonical back-splice sites from isoCirc consensus sequence to reference genome alignment.

a 20-bp windows used for identifying putative canonical back-splice sites from minimap2 alignment of isoCirc consensus sequence to reference genome. At either the start or end coordinate of the minimap2 alignment, a 20-bp window with 10 bp on each side of the coordinate was used to extract the reference genome sequence, which was then used to search for putative canonical back-splice sites.

b Global alignment around putative canonical back-splice sites. If a pair of putative canonical back-splice sites was found in the two 20-bp windows, two 10-bp exonic sequences flanking the canonical back-splice sites were extracted from the reference genome sequence, and re-aligned to the corresponding segment within the isoCirc consensus sequence via a global sequence alignment. The match, mismatch, gap-opening, and gap-extension scores were set as 2, -4, -6, and -2, respectively.

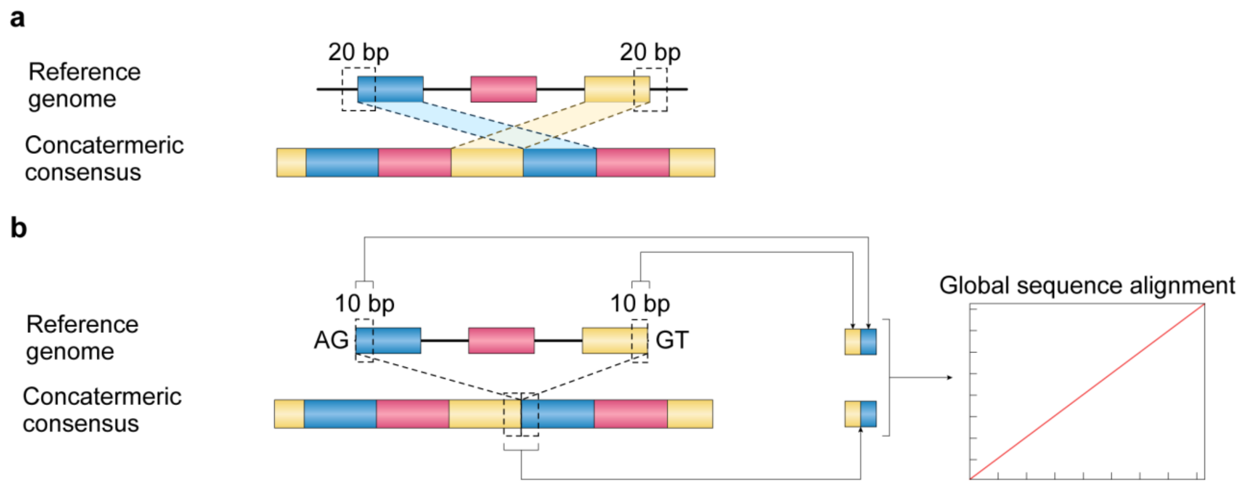

Supplementary Table 1. Read-count, back-splice junction (BSJ), and full-length isoform statistics for circular RNAs (circRNAs) obtained by isoCirc from HEK293 cell line and 12 human tissue samples. High-confidence BSJs obtained by isoCirc were compared with known BSJs in two circRNA databases: circBase and MiOncoCirc.

Sample ID	Description	Overall read count statistics			High-confidence BSJ (hcBSJ) statistics						Full-length isoform statistics		
		Total read count	Reads w/ consensus calling	Reads w/ consensus mapping	Total read count	Unique hcBSJs		Known hcBSJs in circBase	Known hcBSJs in MiOncoCirc	Known hcBSJs in both databases	Total read count	Unique isoforms	
						Read count ≥ 1	Read count ≥ 2					Read count ≥ 1	Read count ≥ 2
Nano-43	HEK293; biological sample 1, technical replicate 1	2,826,759	2,383,764	1,289,077	100,707	48,275	10,614	10,891	18,539	9,845	69,942	41,095	6,929
Nano-44	HEK293; biological sample 1, technical replicate 2	3,023,085	2,559,699	1,384,812	108,973	51,346	11,471	11,193	19,270	10,108	76,179	44,351	7,634
Nano-45	HEK293; biological sample 1, technical replicate 3	4,236,848	3,545,547	1,926,457	148,622	66,194	15,055	12,926	23,122	11,597	103,337	57,763	10,298
Nano-58	HEK293; biological sample 2; technical replicate 1	3,767,164	3,068,885	1,528,147	148,723	61,465	15,602	13,394	25,165	12,053	104,011	54,383	10,807
Nano-59	HEK293; biological sample 2, technical replicate 2	3,816,000	3,102,952	1,549,311	151,037	63,256	15,749	13,493	25,496	12,128	105,614	56,036	10,891
Nano-60	HEK293; biological sample 2; technical replicate 3	2,649,639	2,131,761	1,067,188	103,975	47,434	11,338	11,502	20,639	10,424	72,437	41,120	7,619
Human_adipose	Human adipose tissue	8,947,349	7,558,710	773,140	52,306	28,319	5,676	6,522	11,070	5,903	36,022	24,142	3,589
Human_adrenal_gland	Human adrenal gland tissue	12,414,453	9,042,321	1,985,850	213,677	60,685	18,826	13,775	26,552	12,279	127,340	50,355	12,360

Human_blood	Human blood, peripheral leukocytes	11,216,825	8,384,246	1,500,157	673,032	76,971	33,045	15,965	35,917	13,749	386,562	66,544	24,393
Human_brain	Human brain tissue	8,515,226	6,812,087	1,553,524	415,209	85,468	32,751	16,571	40,877	14,858	268,745	80,100	25,793
Human_heart	Human heart tissue	6,872,058	4,976,966	534,802	74,279	28,648	8,373	8,217	15,137	7,526	43,018	22,881	5,043
Human_kidney	Human kidney tissue	9,225,197	6,936,283	1,724,736	220,862	63,443	20,502	13,936	27,472	12,440	126,545	51,781	12,970
Human_liver	Human liver tissue	8,215,347	5,975,244	735,291	91,869	29,168	9,493	8,741	14,355	7,809	59,594	23,045	6,178
Human_lung	Human lung tissue	16,987,237	10,456,427	1,445,755	140,203	48,333	14,375	11,827	22,006	10,589	82,984	40,107	9,272
Human_prostate	Human prostate tissue	7,564,799	5,298,337	660,581	42,289	22,417	5,051	6,365	10,579	5,810	27,545	18,312	2,981
Human_skeletal_muscle	Human skeletal muscle tissue	7,322,202	6,055,361	418,192	78,114	24,359	8,021	7,825	13,230	7,004	47,948	19,261	5,166
Human_smooth_muscle	Human smooth muscle tissue	6,151,474	4,865,671	786,269	113,463	40,706	12,000	11,600	20,820	10,447	68,950	32,574	7,482
Human_testis	Human testis tissue	7,269,994	5,636,134	2,149,892	470,811	136,422	45,357	18,532	46,760	16,330	284,158	115,348	30,999

Supplementary Table 2. Comparison of circRNA read ratios between short-read and isoCirc long-read datasets of HEK293 cells. Paired-end Illumina sequencing data with 101-bp read length were generated from RNase R-treated libraries (R1, R2, R3) and poly(A)-selected libraries (S1, S2, S3). Data for the six isoCirc datasets of HEK293 cells from Supplementary Table 1 are shown for comparison. The circRNA read ratio was calculated as the read count for BSJs divided by the total read count.

Sample ID	Biological replicate	Technical replicate	Library	Read counts for BSJs	Total read count	circRNA read ratio
S1	1	N/A	poly(A) + Illumina	774	71,910,059	0.001%
S2	2	N/A	poly(A) + Illumina	877	74,451,518	0.001%
S3	3	N/A	poly(A) + Illumina	635	66,249,297	0.001%
R1	1	N/A	RNase R + Illumina	31,710	70,284,760	0.045%
R2	2	N/A	RNase R + Illumina	27,138	56,542,019	0.048%
R3	3	N/A	RNase R + Illumina	27,841	62,545,682	0.045%
Nano-43	1	1	isoCirc	100,707	2,826,759	3.563%
Nano-44	1	2	isoCirc	108,973	3,023,085	3.605%
Nano-45	1	3	isoCirc	148,622	4,236,848	3.508%
Nano-58	2	1	isoCirc	148,723	3,767,164	3.948%
Nano-59	2	2	isoCirc	151,037	3,816,000	3.958%
Nano-60	2	3	isoCirc	103,975	2,649,639	3.924%

Supplementary Table 3. Statistics of circRNA BSJs detected from 8 common human tissues shared between published short-read datasets (Ji et al., 2019, accession number: BIGD ID: PRJCA000751) and isoCirc long-read datasets. In both datasets (Ji et al. (2019) and isoCirc), a read-count cutoff of ≥ 2 reads was used for inclusion of a circRNA in a tissue dataset.

Human tissue	Published short-read datasets (Ji et al., 2019)		isoCirc long-read datasets	
	Number of circRNA BSJs	Total read count	Number of circRNA BSJs	Total read count
Brain	32,350	62,305,388	32,751	8,515,226
Testis	27,831	96,171,912	45,357	7,269,994
Kidney	10,387	90,374,780	20,502	9,225,197
Prostate	8,464	97,603,732	5,051	7,564,799
Liver	7,031	87,582,772	9,493	8,215,347
Heart	7,740	89,736,120	8,373	6,872,058
Lung	6,474	95,271,252	14,375	16,987,237
Skeletal muscle	4,721	87,075,140	8,021	7,322,202

Supplementary Table 4. Sample information and isoCirc sequencing yield for 12 human tissue total RNA samples.

Sample ID	Item description (from Clontech)	Lot Number	Sample information (from Clontech)	FASTQ yield (bp)
Human_lung	Human Lung Total RNA (50 µg)	1807122A	Normal human lung pooled from 3 Asian men, ages: 24, 27, 41 y; cause of death: N/A	35,798,514,970
Human_adrenal_gland	Human Adrenal Gland Total RNA (50 µg)	1703001	Normal adrenal glands pooled from 4 Asian, Caucasian, African American men/women, ages: 43-88 y	45,770,480,898
Human_liver	Human Liver Total RNA (50 µg)	1703002	Normal livers pooled from 4 Asian, Caucasian men/women, ages: 26-78 years, cause of death: N/A	32,172,989,660
Human_kidney	Human Kidney Total RNA (50 µg)	1807151	Normal human kidney pooled from one 26 yo man, two women (63, 83 y), Asian, Caucasian, cause of death: N/A	34,425,257,933
Human_adipose	Human Adipose Tissue Total RNA (50 µg)	636558	Normal human adipose tissue pooled from 18 Caucasian men/women, ages: 22-61 years; cause of death: sudden death	32,413,725,595
Human_skeletal_muscle	Human Skeletal Muscle Total RNA (50 µg)	1612010	Normal skeletal muscle pooled from Asian, Caucasian men/women, ages: 30, 44, 86 y, cause of death: N/A	28,624,651,765
Human_smooth_muscle	Human Smooth Muscle Total RNA (50 µg)	1809103	Normal human smooth muscles pooled from 3 Asian men/women, ages: 21-66 y; cause of death: N/A	28,312,939,703
Human_prostate	Human Prostate Total RNA (50 µg)	1704036A	Normal prostates pooled from 12 Caucasian men, ages: 20-58 y; cause of death: sudden death	22,465,223,103
Human_heart	Human Heart Total RNA (50 µg)	1808022A	Normal human hearts pooled from 3 Caucasian men, ages: 30-39 y; cause of death : trauma	29,528,199,093
Human_brain	Human Brain Total RNA (50 µg)	1812054	Normal human brain (whole) pooled from 3 Asian men, ages: 27-29 y; cause of death: N/A	34,025,701,621
Human_testis	Human Testis Total RNA (50 µg)	1402004	Normal human testes pooled from 7 Asian, Caucasian men, ages: 24-87 y	34,563,956,430
Human_blood	Human Blood, Peripheral Leukocytes Total RNA (10 µg)	1812056	Normal human peripheral leukocytes pooled from 4 men, 1 woman, Black, Hispanic, Caucasian, ages: 30-81 y	32,808,462,079

Supplementary Methods

Details of the isoCirc computational pipeline to identify high-confidence BSJs and full-length circRNA isoforms.

Consensus calling. Tandem repeats in each long read were detected by using Tandem Repeats Finder (TRF, version 4.0.9)[1] (https://tandem.bu.edu/trf/trf.html), with the following recommended settings: *TRF /path/to/long_reads 2 7 7 80 10 100 2000 -h -ngs > /path/to/output.* For each long read, TRF may report multiple tandem repeats (consensus sequences) with different repeat pattern sizes from different segments within the read. The consensus sequence corresponding to a tandem repeat was filtered out if its length was shorter than 30 bp or its repeat copy number within the long read was less than 2. For all consensus sequences that passed these filters, two copies of the same consensus sequence were concatenated and used for downstream analyses (**Fig. 1b**).

Mapping of consensus sequences. The concatemeric copies of the consensus sequences were mapped to the human reference genome (GRCh37/hg19) using minimap2 (version 2.17)[2] with the following settings: *minimap2 -ax splice -ub --MD --eqx /path/to/ref_fa /path/to/cons_fa -t 16 > /path/to/output_sam.* As reverse transcription of a circRNA template could initiate from any random position with a full-length circRNA, we used these concatemeric copies to ensure that the sequence of the full-length circRNA was fully contained in the sequence used for alignment to the human reference genome (**Fig. 1b**).

Selecting the optimal alignment record per consensus sequence. For each consensus sequence called by TRF from a long read, isoCirc filtered out its alignment records with low mapping quality, and then selected the optimal alignment record. For each alignment record, we calculated the ratio of mapped bases (*mapped_ratio*) as the number of mapped bases divided by the length of the consensus sequence (consensus length, in bp). We also calculated the ratio of identically mapped bases (*identical_ratio*) as the number of identical bases divided by the consensus length. Because the concatemers used for alignment contained two copies of the consensus sequence, *mapped_ratio* may be larger than 1.0 when the aligner included more bases on either end of the circRNA template to achieve a higher alignment score. We discarded any alignment record with *mapped_ratio* > 1.1 or < 0.9, or with *identical_ratio* < 0.75. Alignment records passing both criteria were considered as high-quality alignment records. Consensus sequences without any high-quality alignment records were removed from downstream analyses. If a consensus sequence had multiple high-quality alignment records, the alignment record with the highest alignment score as calculated by minimap2 was selected as the optimal alignment record. We also removed a consensus sequence from downstream analyses if the mapped genomic location of any of its high-quality alignment records was neither on autosomes (chromosome 1 to 22) nor on sex chromosomes (chromosome X, Y).

Selecting the optimal consensus sequence per long read. As mentioned above, TRF may detect multiple consensus sequences from each long read. To select the optimal consensus sequence and its optimal alignment record for each long read, we calculated five measures for each consensus sequence:

1. ***bsjAlignScore*** - *Alignment score between consensus sequence and reference genome sequence for the 20-bp exonic sequence flanking the canonical BSJ (10-bp each side)*. To calculate this measure, at either the start or end coordinate of the minimap2 alignment of consensus sequence to reference genome, a 20-bp window with 10-bp on each side of the coordinate was used to extract the reference genome sequence (**Supplementary Fig. 23a**), which was then searched for the occurrence of canonical back-splice sites (GT-AG/GC-AG/AT-AC). If annotated FSJs (Ensembl GRCh37.87) with canonical splice sites were also found in the minimap2 alignment, only putative canonical back-splice sites with the same strandness as the FSJs were considered. For any pair of putative canonical back-splice sites found in the two 20-bp windows, two 10-bp exonic sequences flanking the back-splice sites were extracted from the reference genome sequence, and re-aligned to the corresponding segment within the consensus sequence via a global sequence alignment (**Supplementary Fig. 23b**). The pair of putative canonical back-splice sites with the highest alignment score was selected as the canonical BSJ for the given consensus sequence. If multiple pairs of putative canonical back-splice sites shared the same highest alignment score, isoCirc selected the sites closest to the start and end coordinates of the minimap2 alignment as the canonical BSJ. In the rare event of another tie, the canonical BSJ was selected as the pair of putative canonical back-splice sites that was the leftmost in the downstream 20-bp window, or the leftmost in the upstream 20-bp window if there was a tie in the downstream 20-bp window. Finally, *bsjAlignScore* of each consensus sequence was defined as the alignment score between consensus sequence and reference genome sequence for the 20-bp exonic sequence flanking the canonical BSJ.

2. ***clusterLenFrac*** - *Length fraction of consensus sequence cluster.* To calculate this measure, all similar (ratio of identical bases ≥ 0.8) consensus sequences were first clustered together. Within each cluster, the length fraction of each consensus sequence was defined as the proportion of the entire long read covered by the tandem repeat that was detected by TRF and used to derive the given consensus sequence. *clusterLenFrac* was defined as the sum of all length fractions of consensus sequences in the cluster, and the resulting value was attributed to all individual consensus sequences in the cluster. This measure was designed to address the situation that multiple copies of the circRNA template within an isoCirc long read may be detected by TRF as distinct tandem repeats (and, thus, distinct consensus sequences), due to the high error rate of nanopore sequencing.
3. ***alignScore* -** *Alignment score of consensus sequence to reference genome sequence, from minimap2 alignment file*.
4. ***alignED* -** *Edit distance between consensus sequence and reference genome sequence, from minimap2 alignment file*.
5. ***bsjKeyAlignScore* -** *Alignment score between consensus sequence and reference genome sequence for the 4-bp exonic sequence flanking the canonical BSJ (2-bp each side).*

These five measures were applied sequentially in a multi-tiered manner, such that we only used lower-tier measures in case of a tie with higher-tier measures. Sequentially, the consensus sequence with the highest *bsjAlignScore*, or highest *clusterLenFrac*, or highest *alignScore*, or lowest *alignED*, or highest *bsjKeyAlignScore* was selected as the optimal consensus sequence for a given long read.

Identifying high-confidence BSJs. From the selected optimal consensus sequences of isoCirc long reads, false-positive circRNAs were removed by applying several BSJ-related filters to select high-confidence BSJs. A BSJ was considered to be 'high-confidence' if both its donor and acceptor back-splice sites were catalogued in the gene annotation file (Ensembl GRCh37.87). For BSJs with either an unknown donor or acceptor back-splice site, we applied more stringent filters, and such a BSJ was considered high-confidence only if at least one of its supporting isoCirc long reads' alignments met all of the following criteria:

1. The BSJ's splice site dinucleotide motif was GT-AG.
2. The alignment of the 20-bp exonic sequence flanking the BSJ (10-bp each side) contained no more than 1 mismatch, insertion, or deletion combined.
3. The alignment of the 4-bp exonic sequence flanking the BSJ (2-bp each side) did not contain any mismatch, insertion, or deletion.
4. The distance between the genomic coordinates of the two back-splice sites was no less than a user-defined threshold (default set as 150 bp).

Only circRNAs with a high-confidence BSJ were considered as bona fide circRNAs and included in the isoCirc output file.

Identifying full-length circRNA isoforms. All identified circRNAs with identical coordinates of BSJ/FSJs were pooled together into a unique putative circRNA isoform. Next, isoCirc identified full-length circRNA isoforms in which the BSJ and all FSJs were high-confidence. An FSJ was considered to be 'high-confidence' if both its donor and acceptor forward-splice sites were catalogued in the gene annotation file (Ensembl GRCh37.87). For FSJs with either an

unknown donor or acceptor forward-splice site, we applied more stringent filters, and the FSJ was considered high-confidence only if at least one of its supporting isoCirc long reads' alignments met all of the following criteria:

1. The FSJ's splice site dinucleotide motif was GT-AG/GC-AG/AT-AC.
2. The length of the flanking exon on either side of the FSJ was no shorter than 10 bp.
3. The alignment of the 20-bp exonic sequence flanking the FSJ (10-bp each side), extracted from the minimap2 alignment record, contained no more than 1 mismatch, insertion, or deletion combined.
4. The alignment of the 4-bp exonic sequence flanking the FSJ (2-bp each side), extracted from the minimap2 alignment record, did not contain any mismatch, insertion, or deletion.

CircRNAs in which the BSJ and all FSJs were high-confidence and had the same strandness were considered to be full-length circRNA isoforms. Single-exon circRNAs (with no FSJ) were considered to be full-length circRNA isoforms if they had a high-confidence BSJ.

Supplementary References

1. Benson, G. Tandem repeats finder: a program to analyze DNA sequences. *Nucleic Acids Res* **27**, 573-80 (1999).
2. Li, H. Minimap2: pairwise alignment for nucleotide sequences. *Bioinformatics* **34**, 3094-3100 (2018).
