# Supplementary material for: isoCirc catalogs full-length circular RNA isoforms in human transcriptomes
Source: Nat Commun. 2021 Jan 12;12:266. doi: 10.1038/s41467-020-20459-8 (PMC7803736; doi:10.1038/s41467-020-20459-8)
Supplement: Supplementary file 3 — Reporting Summary [file 41467_2020_20459_MOESM3_ESM.pdf]

nature research

Corresponding author(s): Yi Xing

Last updated by author(s): Nov 13, 2020

Reporting Summary

Nature Research wishes to improve the reproducibility of the work that we publish. This form provides structure for consistency and transparency in reporting. For further information on Nature Research policies, see Authors & Referees and the Editorial Policy Checklist.

Statistics

For all statistical analyses, confirm that the following items are present in the figure legend, table legend, main text, or Methods section.

n/a	Confirmed	
☐	☒	The exact sample size (*n*) for each experimental group/condition, given as a discrete number and unit of measurement
☐	☒	A statement on whether measurements were taken from distinct samples or whether the same sample was measured repeatedly
☐	☒	The statistical test(s) used AND whether they are one- or two-sided<br>*Only common tests should be described solely by name; describe more complex techniques in the Methods section.*
☒	☐	A description of all covariates tested
☐	☒	A description of any assumptions or corrections, such as tests of normality and adjustment for multiple comparisons
☐	☒	A full description of the statistical parameters including central tendency (e.g. means) or other basic estimates (e.g. regression coefficient) AND variation (e.g. standard deviation) or associated estimates of uncertainty (e.g. confidence intervals)
☐	☒	For null hypothesis testing, the test statistic (e.g. *F*, *t*, *r*) with confidence intervals, effect sizes, degrees of freedom and *P* value noted<br>*Give P values as exact values whenever suitable.*
☒	☐	For Bayesian analysis, information on the choice of priors and Markov chain Monte Carlo settings
☒	☐	For hierarchical and complex designs, identification of the appropriate level for tests and full reporting of outcomes
☐	☒	Estimates of effect sizes (e.g. Cohen's *d*, Pearson's *r*), indicating how they were calculated

Our web collection on statistics for biologists contains articles on many of the points above.

Software and code

Policy information about availability of computer code

Data collection	Guppy base-calling software (version 2.1.3) was used to perform base-calling from raw nanopore long-read data (fast5 format).
Data analysis	Tandem Repeats Finder (TRF, version 4.0.9) was used to detect tandem repeats in each long read. minimap2 (version 2.1.7) was used to map concatemeric copies of consensus sequences to the GRCh37/hg19 reference genome. BWA-MEM (version 0.7.17-r1188) was used to align paired-end Illumina sequencing data to the GRCh37/hg19 reference genome. CIRI2 (version 2.0.6) was used to identify circRNA backsplice junctions from alignment records of GRCh37/hg19 reference genome. Source code for the isoCirc pipeline (written in Python/R/Shell) is accessible on GitHub at https://github.com/Xinglab/isoCirc. Details regarding external software settings or custom algorithms used in the isoCirc pipeline can be found in the Methods section and Supplementary Methods section.

For manuscripts utilizing custom algorithms or software that are central to the research but not yet described in published literature, software must be made available to editors/reviewers. We strongly encourage code deposition in a community repository (e.g. GitHub). See the Nature Research guidelines for submitting code & software for further information.

Data

Policy information about availability of data

All manuscripts must include a data availability statement. This statement should provide the following information, where applicable:

- Accession codes, unique identifiers, or web links for publicly available datasets
- A list of figures that have associated raw data
- A description of any restrictions on data availability

Raw data (fastq files) and processed data (abundance measurements) for Illumina short-read and nanopore long-read sequencing of circRNAs in total RNA extracted from HEK293 cells (6 replicates), and nanopore long-read sequencing of circRNAs in total RNA extracted from 12 human tissues, were uploaded to GEO under accession number GSE141693 (https://www.ncbi.nlm.nih.gov/gds/?term=GSE141693). The catalog of full-length circRNA isoforms can be accessed at https://genome.ucsc.edu:/s/xinglab_chop/isoCirc. Annotations for linear transcripts were extracted from the Ensembl GRCh37.87 gene annotation file (ftp://ftp.ensembl.org/pub/grch37/release-87/gtf/homo_sapiens/Homo_sapiens.GRCh37.87.chr_patch_hapl_scaff.gtf.gz). Annotations for repetitive elements from

RepeatMasker were retrieved from the UCSC Table Browser (https://genome.ucsc.edu/cgi-bin/hgTables) on 05/04/2019. Annotations for known circRNA backsplice junctions were downloaded from circBase (http://www.circbase.org) and MiOncoCirc (https://mioncocirc.github.io). Short-read circRNA datasets of human tissues were downloaded under accession number PRJCA000751 from BIGD (https://bigd.big.ac.cn/bioproject/browse/PRJCA000751). Median gene-level TPMs by tissue for GTEx V8 short-read RNA-seq data were downloaded from the GTEx portal (https://gtexportal.org/home/datasets).

Field-specific reporting

Please select the one below that is the best fit for your research. If you are not sure, read the appropriate sections before making your selection.

☒ Life sciences ☐ Behavioural & social sciences ☐ Ecological, evolutionary & environmental sciences

For a reference copy of the document with all sections, see nature.com/documents/nr-reporting-summary-flat.pdf

Life sciences study design

All studies must disclose on these points even when the disclosure is negative.

Sample size	For each biological replicate of HEK293 cells, ~6 million cells grown on a 10-cm petri dish were used for RNA extraction, and the yield (>100ug) was sufficient for 3 technical replicates. For each technical replicate, ~20ug total RNA from the same RNA sample was used for isoCirc library generation. For nanopore (ONT) sequencing, ~300 ng of each isoCirc cDNA library was used, and ~20G bp of data were generated per ONT flow cell. In total, 2 biological replicates each with 3 technical replicates were used to evaluate the reproducibility of the isoCirc method, as customary for evaluating new RNA sequencing methods. For human tissues, ~20-25 ug of total RNA per tissue type was used, with sequencing on multiple ONT flow cells to obtain a yield of ~30G bp of data per tissue type. The total RNA sample of each human tissue, purchased from Clontech, was a pooled sample extracted from tissues of multiple donors.
Data exclusions	No data were excluded from this study.
Replication	For HEK293 cells, we prepared 6 libraries from 2 biological replicates, each with 3 technical replicates. Biological replicates were constructed from RNA samples derived from different cell cultures and different RNA extractions. Technical replicates were constructed separately from RNA samples derived from the same cell culture and RNA extraction. All attempts at replication were successful. In total, 2 biological replicates each with 3 technical replicates were used to evaluate the reproducibility of the isoCirc method, as customary for evaluating new RNA sequencing methods.
Randomization	Randomization was not relevant to this study because our study does not involve the assignment of test subjects or treatments.
Blinding	Blinding was not relevant to this study because our study does not involve assignment of test subjects or treatments.

Reporting for specific materials, systems and methods

We require information from authors about some types of materials, experimental systems and methods used in many studies. Here, indicate whether each material, system or method listed is relevant to your study. If you are not sure if a list item applies to your research, read the appropriate section before selecting a response.

Materials & experimental systems

n/a	Involved in the study	
☒	☐	Antibodies
☐	☒	Eukaryotic cell lines
☒	☐	Palaeontology
☒	☐	Animals and other organisms
☒	☐	Human research participants
☒	☐	Clinical data

Methods

n/a	Involved in the study	
☒	☐	ChIP-seq
☒	☐	Flow cytometry
☒	☐	MRI-based neuroimaging

Eukaryotic cell lines

Policy information about cell lines

Cell line source(s)	Human embryonic kidney cells (HEK293, ATCC #CRL-1573).
Authentication	Genomic DNA of HEK293 cells was authenticated by STR profiling.
Mycoplasma contamination	Cells were confirmed to be mycoplasma-free by the Lonza MycoAlert assay.
Commonly misidentified lines (See ICLAC register)	No commonly misidentified cell lines were used in this study.
